# Harnessing Polymeric Dissolvable Microneedles: Precision Delivery of Therapeutics for Oral Ulcers

**DOI:** 10.1021/acsomega.5c05654

**Published:** 2025-11-19

**Authors:** Maria Nison, Megha Kotian, Vasudev R. Pai, Sony Priyanka Bandi, Popat Mohite, Deepanjan Datta

**Affiliations:** † Department of Pharmacognosy, Manipal College of Pharmaceutical Sciences, 76793 Manipal Academy of Higher Education, Manipal, Karnataka State 576 104, India; ‡ Department of Pharmacy, Birla Institute of Technology and Science (BITS) Pilani, Hyderabad Campus, Hyderabad, Telangana State 500 078, India; § Department of Chemistry, AETs St. John Institute of Pharmacy and Research, Palghar, Maharashtra 401 404, India; ∥ Department of Pharmaceutics, Manipal College of Pharmaceutical Sciences, Manipal Academy of Higher Education, Manipal, Karnataka State 576 104, India

## Abstract

Oral ulcers are a prevalent and recurrent condition that significantly impacts the patient’s quality of life by causing pain, discomfort, and difficulty in eating, speaking, and brushing their teeth. These ulcers can be triggered by diverse etiologies, from trauma to autoimmune disorders, which complicate their management, and they often require targeted treatment to promote healing and alleviate pain. Conventional treatments, such as corticosteroids, nonsteroidal anti-inflammatory drugs (NSAIDs), and topical analgesics, among others, rely on synthetic medications that provide temporary relief and require frequent reapplication, thereby limiting their effectiveness. Thus, the management of oral ulcers necessitates the need for innovative therapeutic approaches. Recent advancements in nanotechnology and transdermal drug delivery have led to the development of dissolving microneedles (MNs) loaded with nanotherapeutics for delivering active therapeutics directly to affected oral tissues as a novel strategy for treating oral ulcers. Dissolving MNs offer a minimally invasive, painless, and efficient method for delivering therapeutic agents directly to the ulcer site, enhancing drug bioavailability, and ensuring sustained release. These biodegradable MNs dissolve upon application, eliminating the need for repeated drug application and thereby improving patient compliance. The synergistic approach of nanotherapeutics encapsulated within dissolving MNs, including nanoparticles, exosomes, and liposomes, among others, effectively addresses the multifactorial nature of oral ulcers by ensuring localized treatment and mitigating the systemic side effects associated with conventional drug delivery methods. Taken together, this review aims to integrate the current knowledge of these innovative technologies while highlighting future directions for research and clinical applications in treating oral ulcers.

## Graphical abstract

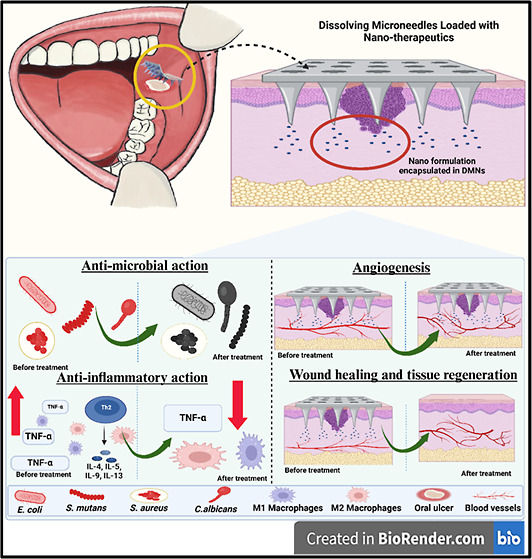

## 1 Introduction

Oral or aphthous ulcers are among the most common oral mucosal diseases affecting 20–25% of the global population across all ages and genders, with a recurrence rate of up to 50%.^1–4^ They are defined as a break in the mucous membrane of the oral cavity marked by numerous small, spherical, or oval ulcers with narrow borders, where there is damage to both the epithelium and lamina propria.^5–7^ They present as erythematous lesions with a yellowish-gray base, often leading to tissue disintegration and necrosis.^3,8^ These lesions vary in size and severity, with minor ulcers healing in 7–10 days and more severe cases lasting weeks or months.^9,10^

Oral ulcer formation is driven by several interrelated biological pathways, with immune-mediated and inflammatory mechanisms playing central roles.^11^ The development of oral ulcers involves a complex interplay of immune-mediated mechanisms, mucosal barrier disruption, and inflammatory responses.^12^ Typically, the process begins with a triggering event, such as trauma, stress, or infection, that leads to local damage of the oral mucosa.^13^ This is followed by an infiltration of immune cells, notably T lymphocytes and neutrophils, and the release of proinflammatory cytokines such as TNF-α, IL-2, and IL-6, resulting in epithelial cell apoptosis and breakdown of the mucosal barrier. The subsequent exposure of the underlying connective tissue leads to ulcer formation with necrosis and fibrin deposition at the base of the lesion. Genetic predisposition and dysregulation of the local immune response further contribute to the chronicity and recurrence of these ulcers.^1^ Dysregulation of the immune system, particularly involving T-cell-mediated cytotoxicity and the release of proinflammatory cytokines such as TNF-α and interleukins, leads to epithelial cell apoptosis and oral mucosal damage.^14^ Inflammatory cascades further amplify tissue injury through the production of mediators like prostaglandins and reactive oxygen species (ROS).^15^ Disruption of the mucosal barrier due to trauma,^16^ infections,^11,17^ and direct epithelial injury exposes underlying tissues and initiates ulcer development. Nutritional deficiencies, especially of iron,^18,19^ folic acid,^20^ and vitamin B_12_,^21^ and autoimmune diseases such as Behçet’s disease,^22^ gastrointestinal disorders such as Crohn’s disease,^23^ and ulcerative colitis,^24^ compromise mucosal integrity and repair, increasing susceptibility to ulcers. Additionally, microbial infections, certain medications including NSAIDS,^25,26^ β-blockers,^27^ anticancer,^28,29^ antihypertensives,^30^ and psychological stress,^31–33^ can trigger or exacerbate these pathways. Ultimately, the interplay of these immune, inflammatory, barrier, nutritional, and systemic factors underlies the complex pathogenesis of oral ulcers.

Current treatments, like topical corticosteroids,^34,35^ antiseptic mouthwashes,^36^ and analgesics,^37^ can reduce inflammation but often require frequent reapplication and may cause side effects like mucosal thinning or fungal infections.^15,38^ Their effectiveness is limited due to the constant movement of the oral cavity, the flushing action of saliva, and the variable pH environment of the oral mucosa, which can fluctuate between pH 6.2 and 7.8 depending on factors like diet, salivary flow, and oral hygiene.^39,40^ Many patients struggle with adherence, leading to delayed healing and recurrence. Therefore, there is an acute demand for novel therapeutic strategies for treating oral ulcers that are both practical and safe.

To this end, advanced nanoformulations, including nanoparticles, liposomes, nanoemulsions, nanofibers, nanocrystals, nanogels, nanosheets, and semisolid dosage forms such as hydrogels and bioadhesive patches, have shown targeted and sustained drug release at the ulcer site, reducing the need for frequent reapplication and improving patient compliance (Figure 1).^41^ However, ensuring adequate mucoadhesion and prolonged contact time with the ulcer site remains challenging, as these nanocarriers can be easily washed away or diluted by the constant flow of saliva and the viscoelastic mucus overlying ulcers, thereby reducing contact time at the lesion site and impairing drug uptake.^42^ The oral cavity’s pH can swing between 5.5 and 7.5, and salivary enzymes (e.g., amylases, proteases) can destabilize many nanocarriers, leading to premature payload release or carrier breakdown before reaching the target tissue.^43^ Moreover, the intact mucosal epithelium relies on tight junctions to block foreign particles. Nanosystems larger than 200 nm struggle to permeate paracellular spaces, limiting drug penetration into ulcerated tissues.^44^ Overcoming these hurdles often requires hybrid strategies combining nanoformulations with mucoadhesive biopolymers, thermoresponsive gels, or dissolvable MNs, to enhance retention, protect against degradation, and facilitate controlled release.^45–47^

**Figure 1:**
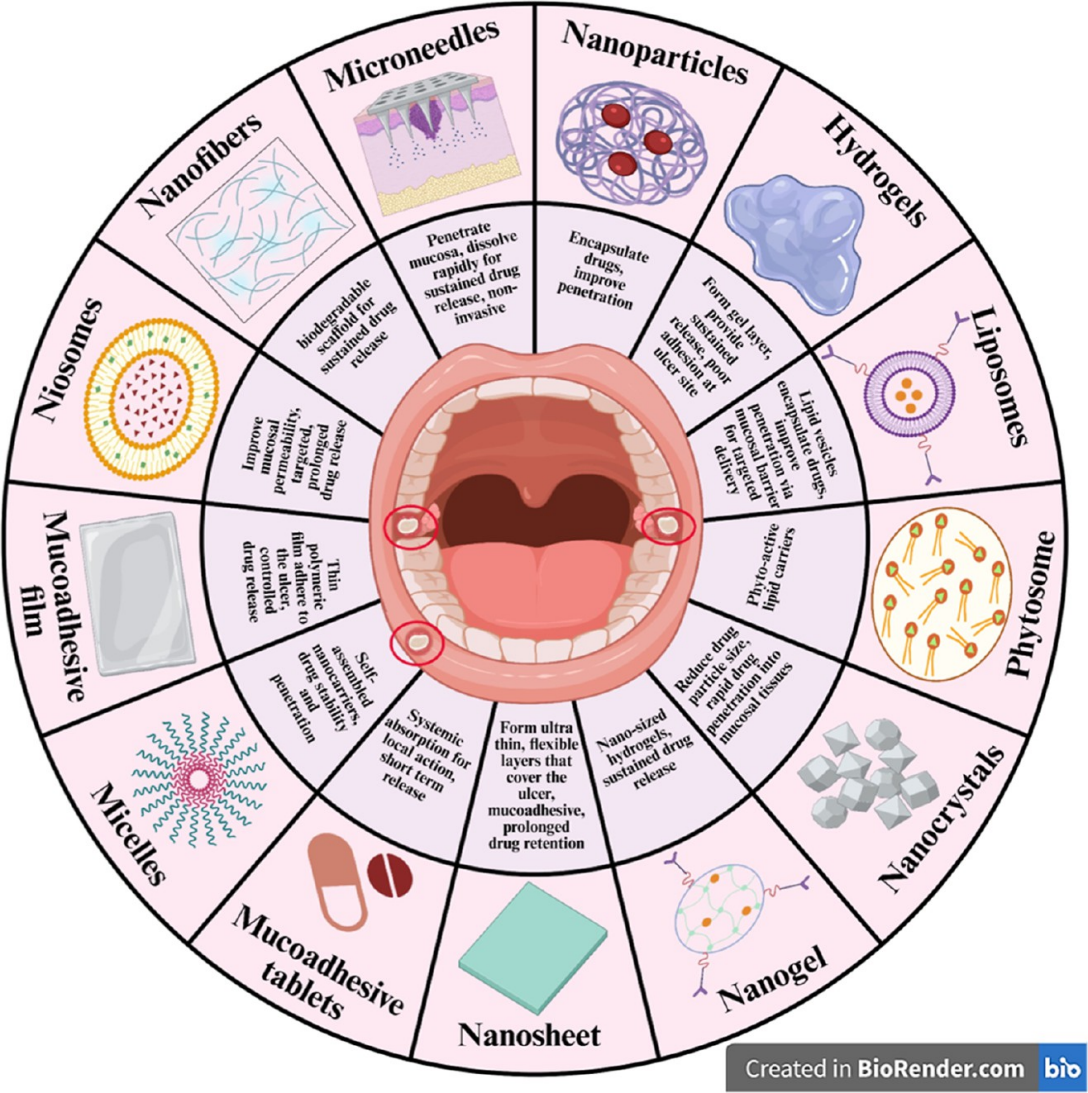
Schematic representation of novel drug delivery systems and their mechanism of action for treating oral ulcers (created with https://biorender.com/).

In recent years, dissolving MNs has emerged as a promising strategy for localized and controlled drug delivery for treating oral ulcers. They offer a promising approach for the treatment of oral ulcers by enhancing drug delivery and stability in a challenging oral environment. Unlike traditional nanoparticles or gels that may suffer from poor retention, instability, or rapid drug erosion due to saliva, dissolving MNs provide direct and sustained drug delivery by penetrating the buccal mucosal layers and dissolve rapidly upon application at the ulcer site.^48^ This ensures enhanced drug bioavailability, prolonged therapeutic effects, which maximizes local drug concentration while reducing systemic side effects.^49^ Their biodegradable nature eliminates the risk of toxicity, improving safety and patient compliance. Moreover, dissolving MNs can encapsulate multiple therapeutic agents^50,51^ or nanobased formulations,^51^ allowing for combination therapy that simultaneously addresses inflammation, infection, and tissue regeneration.^52^ By offering a painless and accessible alternative, dissolving MNs showed the potential to revolutionize oral ulcer management and provide effective therapy.

The synergistic approach of nanoformulations into dissolving MNs represents a significant advancement in oral ulcer treatment, combining the strengths of both technologies to address the limitations of conventional therapies.^53^ Nanoformulations enhance drug stability, bioavailability, and targeted delivery, while minimally invasive dissolving MNs enable precise, painless, and sustained drug administration directly at the ulcer site. This approach overcomes challenges put forward by the conventional treatments for oral ulcers, thereby offering a controlled release of therapeutic agents for prolonged action and improved patient adherence. Clinical and preclinical studies have demonstrated that dissolving MNs loaded with corticosteroids like triamcinolone acetonide and betamethasone, as well as multifunctional combinations incorporating growth factors and antibacterial agents, can significantly enhance ulcer healing, reduce inflammation, and improve patient comfort compared to traditional topical or injectable therapies.^54,55^ Further advancements include the development of HA-based dissolving MNs,^56^ and curcumin-loaded nanoparticles^57^ that have shown enhanced therapeutic efficacy in promoting oral ulcer healing. The synergistic integration of nanotechnology with dissolving MNs offers a promising, minimally invasive approach for treating oral ulcers by enabling the targeted and efficient delivery of therapeutic agents. Studies have demonstrated that incorporating nanomaterials such as mesoporous polydopamine nanoparticles,^45^ zeolitic imidazolate framework-8 (ZIF-8),^58^ exosomes,^51^ and polysaccharide composites into MNs^59^ enhances anti-inflammatory, antibacterial, and tissue-regenerative effects, leading to accelerated healing and improved patient outcomes. By improving drug penetration, minimizing systemic exposure, and optimizing treatment outcomes, these polymeric dissolvable MNs with nanoformulations hold transformative potential for oral ulcer management.

Taken together, in a nutshell, this extensive review article delves into the development and validation of a polymeric dissolvable microneedle platform that delivers therapeutics directly into ulcerated oral mucosa with high precision, minimal invasiveness, and rapid payload release, maintaining its mechanical integrity during application, dissolving fully within minutes, and achieving localized drug concentrations that outperform conventional topical formulations in *in vivo* ulcer models. Importantly, cytocompatibility assays have confirmed their safety for delicate mucosal tissues, and their rapid dissolution circumvents the clearance challenges of the dynamic oral environment. By combining tunable polymer chemistry with patient-friendly design, this approach significantly enhances treatment efficacy, reduces systemic exposure, and boosts compliance through painless administration. These dissolvable polymeric MNs represent a transformative step toward precision, patient-centric therapy for oral ulcers, and potentially a blueprint for next-generation mucosal drug delivery systems.

## 2 The Dynamic Shield: Exploring the Architecture and Roles of the Oral Mucosa

Oral mucosa is the mucous membrane that covers all oral structures except the clinical crowns of the teeth.^60^ It is anatomically divided into three layers: the outermost layer of stratified squamous epithelium, followed by the basement membrane, and the connective tissue composed of the lamina propria and submucosa (Figure 2).^61,62^ The thickness and keratinization of the epithelium, as well as its melanin pigments, and the vascularization of the connective tissue determine the color of the respective area of the oral mucosa.^63^ Depending on its location, the epithelium may be keratinized, parakeratinized, or nonkeratinized.^64^ The lamina propria varies in thickness and supports the epithelium. It is attached to the alveolar bone’s periosteum or interposed over the submucosa, which may differ in different regions of the mouth (e.g., floor of the mouth, soft palate). The submucosa, consisting of connective tissues varying in density and thickness, attaches the mucous membrane to the underlying bony structures. The submucosa contains glands, blood vessels, nerves, and adipose tissue.^65^

**Figure 2:**
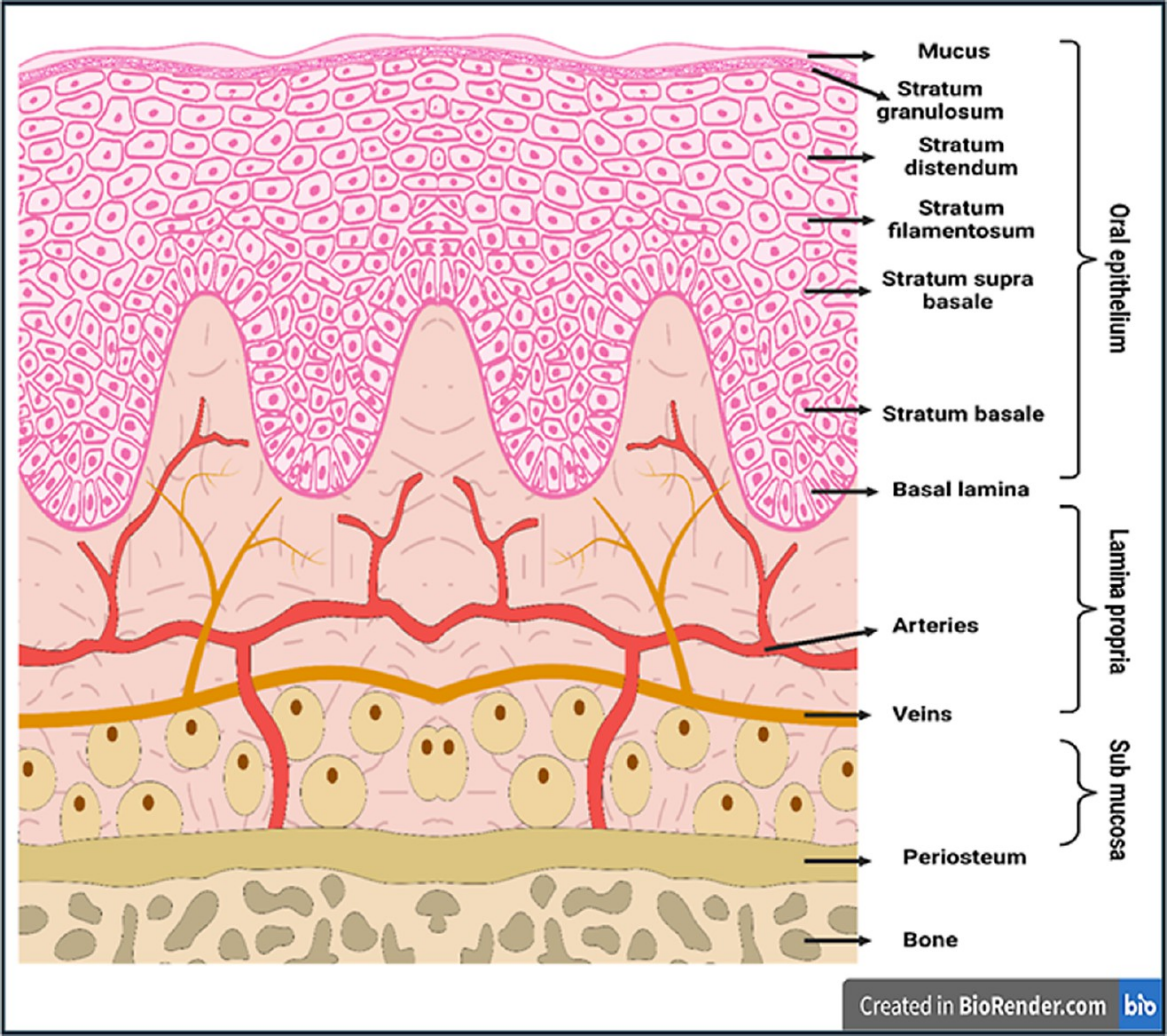
Schematic representation showing the different layers of the oral mucosa (created with https://biorender.com/).

Oral mucosa is classified into three types, based on its major functional types: (1) masticatory mucosa, (2) lining or reflective mucosa, and (3) specialized mucosa.^66^ The masticatory mucosa comprises the free and attached gingiva and the hard palate. Areas of the oral cavity that are constantly exposed to high mechanical stress due to the mastication of food are covered by the masticatory mucosa. This mucosa is attached directly or indirectly (by a fibrous submucosa) to the periosteum of the underlying bone and, thus, is immobile. In adaptation to high mechanical load, the epithelium is moderately thick and keratinized, and numerous long papillae provide a robust attachment to a thick lamina propria.^65^ The epithelium of these tissues is keratinized, and the lamina propria is a dense, thick, and firm connective tissue containing collagen fibers. The hard palate has a distinct submucosa except for a few narrow, specific zones. The dense lamina propria of the attached gingiva is connected to the cementum and periosteum of the bony alveolar process. The lining or reflective mucosa covers the inner parts of the lips, buccal mucosa, cheek, and vestibule, the lateral surfaces of the alveolar process (except the mucosa of the hard palate), the floor of the mouth, the soft palate, and the ventral surface of the tongue.^67^

The lining mucosa is a thin, movable tissue with a relatively thick, nonkeratinized epithelium and a thin lamina propria.^68^ The submucosa comprises mostly thin, loose connective tissue with muscle and collagenous; and elastic fibers, with different areas varying in their structures. The junction of the lining mucosa and the masticatory mucosa is the mucogingival junction, located at the apical border of the attached gingiva, facially and lingually in the mandibular arch and facially in the maxillary arch.

The specialized mucosa covers the dorsal regions of the tongue and the taste buds. The epithelium is nonkeratinized except for the covering of the dermal filiform papillae. Although the oral mucosa shares a similar histological structure with the skin, there are notable differences between them (Figure 3). First, the oral mucosa does not have a cuticle. Second, the mucosal basal cells are rectangular, whereas the skin cells are cylindrical. Third, although the epithelial cells of both evolve from the basal cells, the hierarchy and morphology between the epithelial cells of the mucosa are less regular than that of the skin. Fourth, there are more blood vessels in the lamina propria of the mucosa.

**Figure 3:**
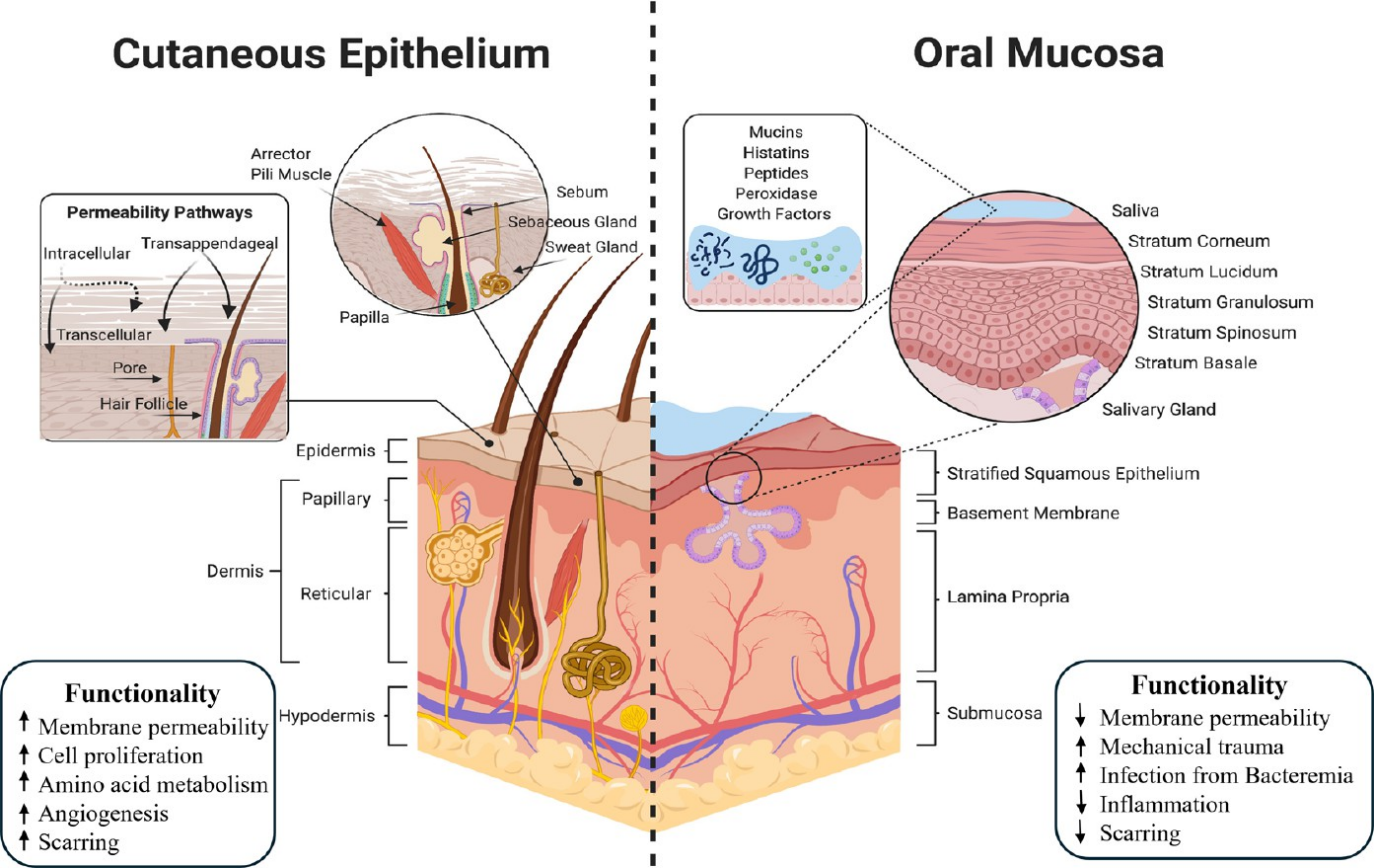
Comparative schematic of skin and oral mucosa. The skin comprises epidermis, dermis, and hypodermis, enabling transdermal transport via transcellular, intercellular, and appendageal routes. Oral mucosa features stratified squamous epithelium with underlying lamina propria and submucosa, supported by saliva-derived bioactive components. Injury increases susceptibility to infection due to diverse oral microflora. Reprinted with permission from ref 69 Copyright 2021, Elsevier.

The thickness and surface area of the oral mucosa are about 500–800 μm and 200 cm^2^, respectively.^70^ It serves as a multifunctional barrier, providing mechanical protection against physical forces during activities such as chewing and speaking while also offering immunological defense against pathogens and harmful substances. Its rich innervation allows for the perception of temperature, pressure, and pain, safeguarding the gastrointestinal tract from potential harm. Notably, the oral mucosa exhibits a permeability that is 4 to 4,000 times greater than that of the skin, facilitating efficient drug absorption.^71^ This permeability varies across different regions, with the sublingual mucosa being the most permeable due to its thin, nonkeratinized epithelium, followed by the buccal and palatal mucosa. The continuous renewal of oral mucosal cells ensures that superficial cells can be shed due to mechanical stress without compromising the integrity of the barrier. These characteristics underscore the oral mucosa’s critical role in protection and sensation and as a viable route for drug delivery.

### Drug Absorption in the Oral Mucosa

The oral mucosa presents a unique and versatile route for both local and systemic drug delivery, owing to its rich vascularization and ability to bypass hepatic first-pass metabolism. However, its heterogeneous nature, characterized by regional variations in thickness, keratinization, and blood supply, significantly influences drug absorption profiles. The epithelium is the primary target for drug delivery in treating most oral mucosal diseases.^72^ Passive diffusion is the primary mode of drug transport across the lipid-rich mucosal epithelium, while certain molecules, such as D-glucose, amino acids, and vitamins, are absorbed via specialized active transport mechanisms.^73^ By rationalizing the oral mucosa into a hydrophobic membrane, Fick’s first law can be used to describe the drug absorption process (eqs 1 and 2)^74^

1 $$D=\frac{PK_{p}}{h}$$

The amount of drug absorbed, *A*, is given by

2 $$A=PCS_{t}=\frac{DK_{p}}{h}CS_{t}$$

where *P* is the permeability coefficient, *A* is the amount of drug absorbed, *D* is the diffusion coefficient of the drug in the oral mucosa, *K*_p_ is the partition coefficient of the drug between the delivery medium and the oral mucosa, *h* is the thickness of the oral mucosa, *C* is the free drug concentration in the delivery medium, *S* is the surface area of the delivery site on the oral mucosa, and *t* is the duration of drug contacting the oral mucosa.

Drug permeability is inversely related to molecular weight, with compounds exceeding 800 Da exhibiting limited transmucosal penetration.^75^ Moreover, the balance between lipophilicity and hydrophilicity is critical; excessive lipophilicity impairs solubility in saliva, while high hydrophilicity restricts membrane permeation. Optimal drug candidates typically exhibit log *P* values between 1.6 and 3.3, ensuring adequate solubility and membrane diffusion.^76^ Stability within the salivary pH range (6.28 ± 0.36) further supports effective absorption.^77^ While log *P* offers insight into lipophilicity, the distribution coefficient (log *D*), which accounts for ionization at physiological pH, provides a more accurate predictor of mucosal permeability. For oral ulcers, local drug delivery via mucoadhesive systems is preferred, enabling direct absorption and enhanced therapeutic efficacy. Formulation parameters such as surface area, contact time, and free drug concentration play a pivotal role in optimizing bioavailability.^78,79^ Interactions within the dosage form that sequester the drug can significantly reduce its availability for absorption.^80^ Additionally, the limited surface area and rapid clearance mechanisms of the oral cavity constrain the maximum deliverable dose, typically a few milligrams per day.

## 3 Oral Ulcers Decoded: A Guide to Their Varied Forms

Oral ulcers are common lesions characterized by the breakdown of the mucosal lining in the oral cavity. They can arise from various causes, including trauma, infections, systemic diseases, or idiopathic factors. Understanding the different types of oral ulcers is crucial for an accurate diagnosis and effective management.

a. Based on duration, oral ulcers are classified as
  i Acute or short-term—persists for not more than 3 weeks. It can recur as aphthous ulcers, traumatic ulcers, herpetic ulcers, and chancres.^62^
  ii Chronic or long-term—continues for weeks and months as a significant aphthous ulcer, malignant ulcer, or some traumatic ulcer (with a persistent traumatic element).^10,80^

b. Based on the presentation, oral ulcers are classified as
  i Single-solitary lesions that result from injury and infection, or can be carcinomas.
  ii Multiple–Multiple lesions are associated with viral infections and autoimmune diseases. It can present with several ulcerations. Intermittent healing and a history of comparable events may be evident in recurrent ulcers. Ulcers can range in size from a few millimeters to several centimeters, and they can sometimes show up with a fever and localized lymphadenopathy.^81^
c. Classification of oral ulcers based on appearance, size, and etiology:

Oral ulcers are common lesions that manifest within the oral cavity, often causing discomfort and posing diagnostic challenges due to their varied presentations. These ulcers can be broadly classified based on their appearance, size, and underlying causes, facilitating a more systematic approach to diagnosis and treatment. Understanding these classifications is crucial for clinicians to differentiate between benign and potentially serious conditions, ensuring that appropriate management strategies are employed. Table 1 provides an overview of the classification of oral ulcers, highlighting their categorization based on appearance, size, and etiology.

**Table 1:** Classification of Oral Ulcers Based on Appearance, Size, and Underlying Causes^62,82,83^

type	description	size	duration	frequency
minor ulcers	small, round, or oval lesions with a white or yellow center and a red border	typically, less than 10 mm in diameter	heals within one to two weeks without scarring	the most common type, accounting for about 80% of mouth ulcers
major ulcers (major aphthous ulcers)	more extensive and more profound than minor ulcers, often with irregular edges	more than 10 mm in diameter	it may take several weeks to months to heal and can leave scars	less common, accounting for about 10% of mouth ulcers
herpetiform ulcers	numerous small ulcers, often in clusters that may merge into larger sores	each ulcer is typically 1–3 mm in diameter	usually heals within one to two weeks without scarring	rare, accounting for about 5–10% of mouth ulcers
traumatic ulcers	caused by physical injury to the mouth, such as biting the cheek, dental work, or abrasive foods	it varies depending on the injury, but it typically heals once the source of trauma is removed	standard, depending on the cause	standard, depending on the cause
drug-induced ulcers	caused by a reaction to certain medications, such as NSAIDs, beta-blockers, or chemotherapy agents	vary depending on the drug and individual response	less common but significant when they occur	less common but significant when they occur
infectious ulcers	caused by infections, such as viral (herpes simplex virus), bacterial, or fungal infections	vary depending on infection and treatment	vary depending on the underlying infection	vary depending on the underlying infection
systemic disease-associated ulcers	linked to systemic illnesses such as systemic lupus erythematosus, Crohn’s disease, Behçet’s disease, and celiac disease	vary depending on the underlying disease and its management	vary depending on the prevalence of the associated condition	vary depending on the prevalence of the associated condition

### 3.1 Molecular Clues: Understanding the Origins of Oral Ulcers

#### 3.1.1 Pathogenesis

The exact pathogenesis of oral ulcers is not well-established. There is strong evidence from histopathological and immunological studies that T-cell-mediated immune responses are implicated in oral ulcers (Figure 4).^84–86^

**Figure 4:**
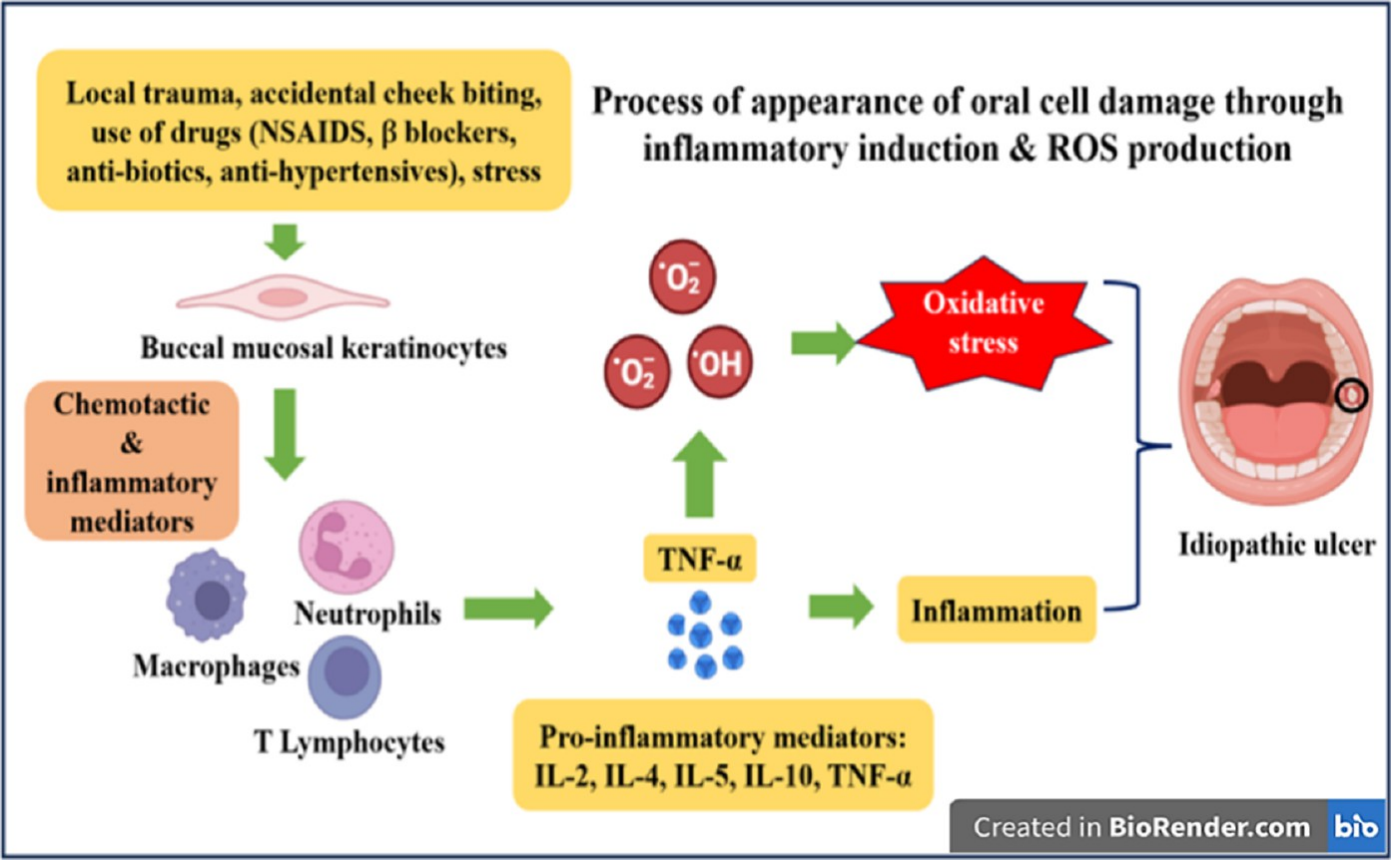
Pathogenesis of oral ulcer. The schematic depicts the cascade leading to oral ulceration: epithelial disruption from trauma or irritants triggers innate immune activation and antigen presentation. Cytokine imbalance, neutrophil infiltration, oxidative stress, and complement activation amplify tissue damage (created with https://biorender.com/).

Three stages are recognized in the development of oral ulcerations (Figure 5).^87,88^ The development and healing of oral ulcers involve a complex interplay of cellular and molecular events. Initially, mononuclear (lymphocytic) cells infiltrate the oral epithelium, leading to focal vacuolation. This is followed by the degeneration of suprabasal epithelial cells and a mononuclear infiltrate in the lamina propria. As the ulcerative stage progresses, there is significant infiltration of mononuclear cells, particularly within the epithelium, accompanied by pronounced edema and epithelial degradation. These changes culminate in the formation of ulcers covered by a fibrinous membrane.^89^

**Figure 5:**
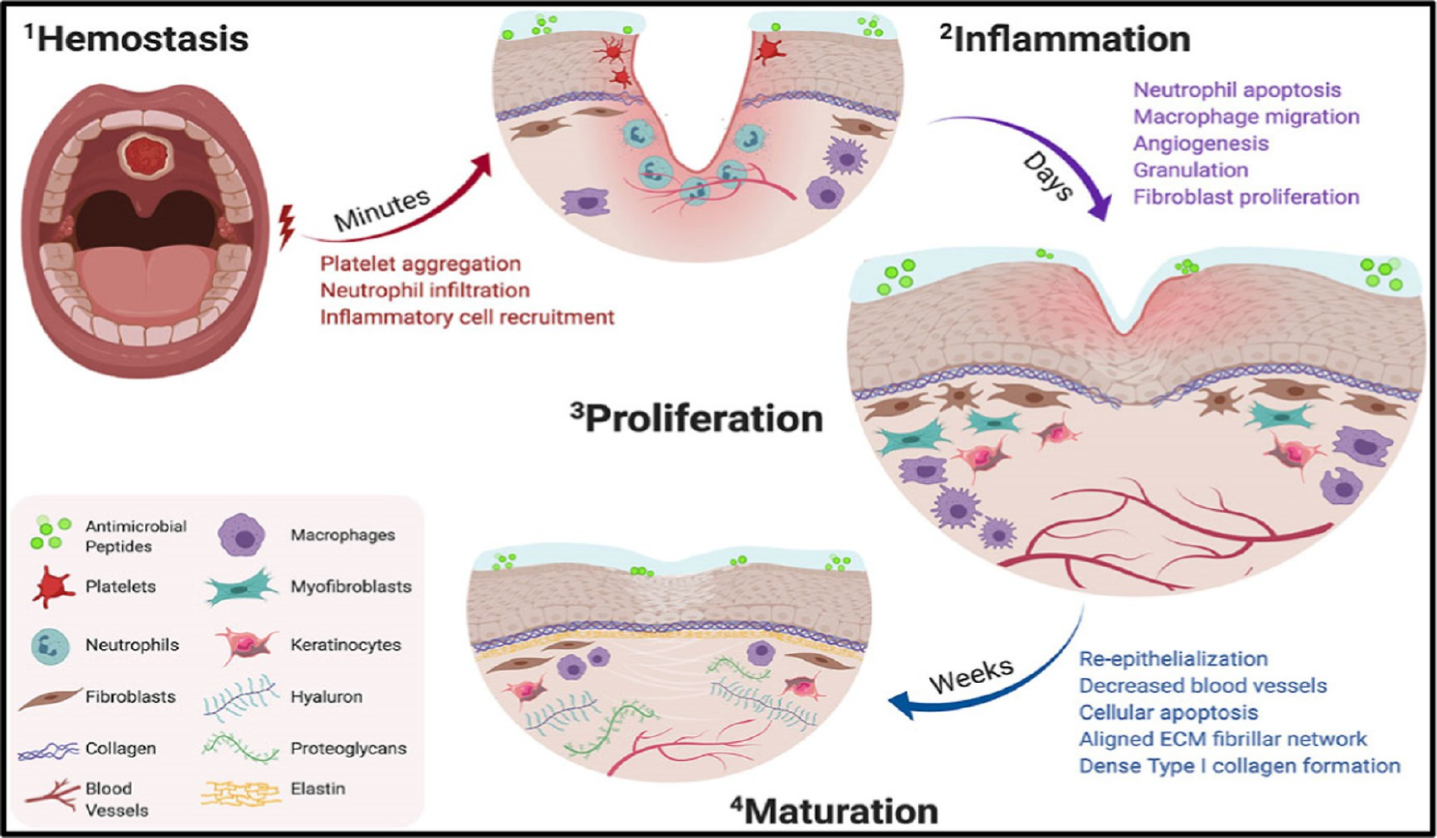
Timeline of oral wound healing and oral mucosal remodeling. The schematic outlines four phases of oral wound healing: hemostasis, inflammation and macrophage activity with cytokine release, proliferation, and maturation. Reprinted with permission from ref 88 Copyright 2024, Frontiers.

The pathogenesis of mouth ulcers is multifactorial, involving trauma, infections, and certain medications that damage the epithelial layer. The body’s response includes inflammation characterized by pain, swelling, and redness. Tumor necrosis factor-α (TNF-α) plays a pivotal role by promoting endothelial cell adhesion and neutrophil chemotaxis, amplifying the inflammatory response. The activation of immune cells, including T-cells and macrophages, contributes to ulcer development, with the release of proinflammatory cytokines like TNF-α and interleukin-1 beta (IL-1β) further exacerbating inflammation. Secondary colonization by bacteria, viruses, or fungi can perpetuate inflammation and delay healing. Healing of oral ulcers follows a structured process:^88^

I Hemostasis: Immediately after injury, blood vessels constrict, and clotting mechanisms activate to halt bleeding, forming a fibrin clot that serves as a temporary matrix for cell migration.
II Inflammation: Within the first few days, neutrophils and macrophages infiltrate the wound site to remove debris and pathogens, releasing inflammatory cytokines to facilitate healing.
III Proliferation: Around days 4 to 7, fibroblasts migrate to the wound bed, depositing extracellular matrix components like collagen. Angiogenesis occurs, forming new blood vessels to supply nutrients, while re-epithelialization begins as keratinocytes cover the wound surface.
IV Remodeling: Starting approximately one week post-injury, collagen fibers reorganize and strengthen, restoring tissue integrity and function over time.

This efficient healing process in the oral mucosa is facilitated by factors such as rich vascularization and the presence of saliva, which contains growth factors and antimicrobial agents. Oral ulcers are related to ROS.^90^ ROS can damage epithelial cells and tissues in the mouth, leading to ulcer formation. ROS can also trigger and amplify the inflammatory response and exacerbate the symptoms of mouth ulcers.^91^ An imbalance in the generation of ROS and antioxidant defenses can result in oxidative stress, which can aid in developing oral ulcers.

#### 3.1.2 Ethiopathogenesis

The precise etiopathogenesis of oral ulcers is not fully disclosed. Their development is multifactorial, involving genetic, mechanical, psychological, dietary, microbial, and immunological factors, as depicted in Figure 6.

**Figure 6:**
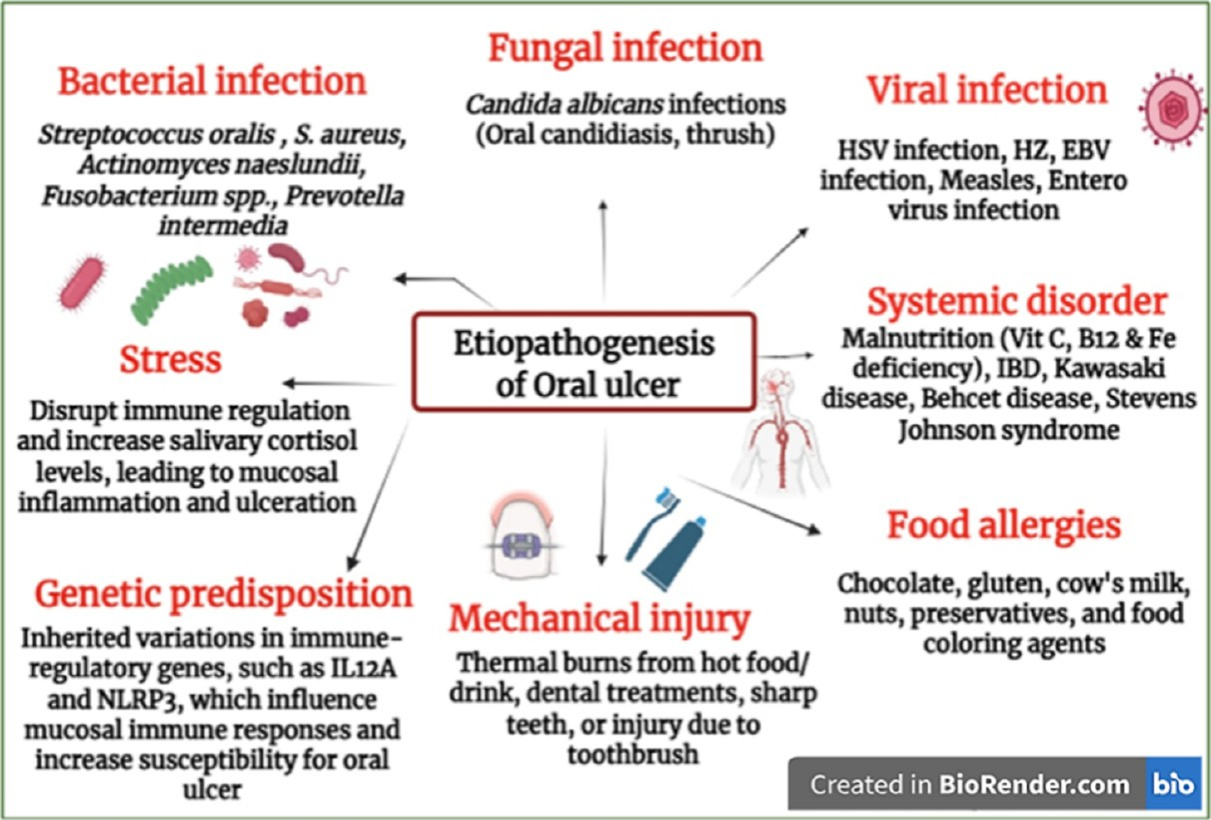
Etiopathogenesis of oral ulcers. The diagram depicts the multifactorial origin of oral ulcers that triggers epithelial disruption, amplifies inflammatory cascades, and impairs healing, leading to ulcer formation and persistence (created with https://biorender.com/).

##### 3.1.2.1 Genetic Predisposition

Genetic susceptibility plays a pivotal role in the etiopathogenesis of oral ulcers, with familial aggregation observed in approximately 24–46% of cases.^92^ Early studies suggested autosomal recessive or polygenic inheritance patterns, further supported by higher concordance rates in monozygotic twins.^93,94^ Individuals with a positive family history, particularly when both parents are affected, exhibit increased risk, earlier onset, and greater severity. Polymorphisms in cytokine-related genes (e.g., IL-1β, IL-6, IL-10, TNF-α) and specific HLA alleles (HLA-B12, HLA-B51, HLA-DR7) have been implicated, although ethnic variability influences these associations.^95,96^ These findings underscore the importance of genetic profiling in understanding disease mechanisms and tailoring preventive or therapeutic strategies.

##### 3.1.2.2 Mechanical Injury

In individuals predisposed to oral ulcers, they frequently arise following mechanical irritation such as dental procedures, sharp teeth, or trauma from toothbrush use.^97^ Although the precise pathophysiology remains unclear, neutrophil elastase has been implicated in posttraumatic aphthous ulcer formation. Epidemiological data indicate a lower prevalence of oral ulcers among smokers, with a protective effect linked to increased mucosal keratinization and reduced susceptibility to injury.^98^ Nicotine and its metabolites may further modulate the inflammatory response by downregulating proinflammatory cytokines (TNF-α, IL-1, IL-6) and upregulating IL-10.^99^ Similar trends have been observed with tobacco use. Local factors, including anesthetic injections and inadequate salivary flow, also contribute to ulcer development by compromising mucosal integrity and increasing antigenic exposure.^100^

##### 3.1.2.3 Stress

Another factor potentially linked to oral ulcers is stress. The patient usually displays elevated stress with the oral ulcer initiation, and various other studies have reported a higher occurrence rate.^101^ There can be genetically determined anxiety or unknown biochemical effects that are responsible for parafunctional habits, including the biting of the cheek and lip, and physical trauma that begins the process of ulceration in individuals vulnerable to it.^102^

##### 3.1.2.4 Food Allergies

Micronutrient deficiencies, particularly iron, folic acid, and vitamin B_12_, have been frequently observed in patients with oral ulcers, though supplementation yields a limited benefit for most. A notable trial by Volkov et al. reported a significant reduction in ulcer frequency and duration with daily vitamin B_12_ supplementation, independent of baseline serum levels.^103^ The role of zinc remains controversial; while some studies link deficiency to ulceration and symptom relief with supplementation, others report inconsistent findings.^104^ Dietary triggers such as chocolate, gluten, dairy, nuts, preservatives, and food additives have been implicated anecdotally, with some patients experiencing improvement upon elimination.^105^ However, controlled studies suggest these effects may be nonspecific or placebo-driven, and the overall impact of dietary habits on ulcer pathogenesis remains inconclusive.^106,107^

##### 3.1.2.5 Bacterial and Viral Infections

The involvement of microbial agents in oral ulcer pathogenesis remains speculative. *Streptococcus oralis* has been associated with elevated antibody titers in affected individuals, though subsequent studies failed to confirm a consistent etiological role.^108^ *Helicobacter pylori* has garnered attention due to its impact on systemic vitamin B_12_ levels; eradication therapy has been linked to reduced aphthous lesions, suggesting an indirect benefit via improved micronutrient absorption rather than direct mucosal involvement.^109^ Notably, a study by Taş et al. demonstrated that eradication of *H. pylori* led to a significant increase in serum vitamin B_12_ levels and a reduction in the number of aphthous lesions.^110^ Several viral agents, including Herpes Simplex Virus (HSV-1 and HSV-2), Varicella-Zoster Virus (VZV), Cytomegalovirus (CMV), Epstein–Barr Virus (EBV), and Adenoviruses, have been investigated through PCR-based detection in ulcerative lesions.^111^ While viral DNA has been sporadically identified, the evidence does not support a direct relationship. Additionally, *Candida albicans* and other oral commensals have been explored for their potential role in mucosal disruption, particularly in immunocompromised individuals, although their contribution to idiopathic aphthous ulcers remains unsubstantiated. Studies by Greenspan et al. further refute the hypothesis of cell-mediated hypersensitivity or antigenic cross-reactivity between microbial antigens and oral mucosa.^112^ Overall, while microbial agents may act as modulators or secondary contributors, the current evidence does not establish a definitive causal link in the etiopathogenesis of oral ulcers.

#### 3.1.3 Immunopathogenesis

The development of oral ulcers is driven by a complex interplay of immune dysregulation, genetic predisposition, and environmental triggers. At their core, oral ulcers arise from immunologic abnormalities that disrupt the balance between proinflammatory and anti-inflammatory pathways, leading to localized tissue damage and impaired healing (Figure 7). The primary mechanism underlying oral ulcers involves a T-cell-mediated immune response.^113^ When the oral mucosa is exposed to triggers such as heat shock proteins, minor trauma, or microbial antigens, antigen-presenting cells activate CD4+ T-helper lymphocytes. These activated T cells release a cascade of proinflammatory cytokines, including interleukin-2 (IL-2), interferon-gamma (IFN-γ), human oral-α, and IL-12, which stimulate the humoral immune response and the secretion of IgE, which recruit additional immune cells to the site of injury.^114^ This inflammatory cascade results in tissue breakdown and ulcer formation. Studies have consistently shown elevated levels of these cytokines in the serum and saliva of affected individuals, further supporting their central role in pathogenesis. A critical feature of oral ulcers is the imbalance between proinflammatory and anti-inflammatory cytokines. While IL-2, TNF-α, and IFN-γ are markedly elevated, anti-inflammatory mediators such as IL-10 are suppressed. This skewed cytokine profile perpetuates inflammation and delays mucosal repair.^115^ Recent research has also highlighted the involvement of Th17 cells and IL-17A, a cytokine that amplifies neutrophil recruitment and exacerbates tissue damage. Elevated IL-17A levels in patients with recurrent ulcers suggest its contribution to chronic inflammation and lesion persistence.^116^

**Figure 7:**
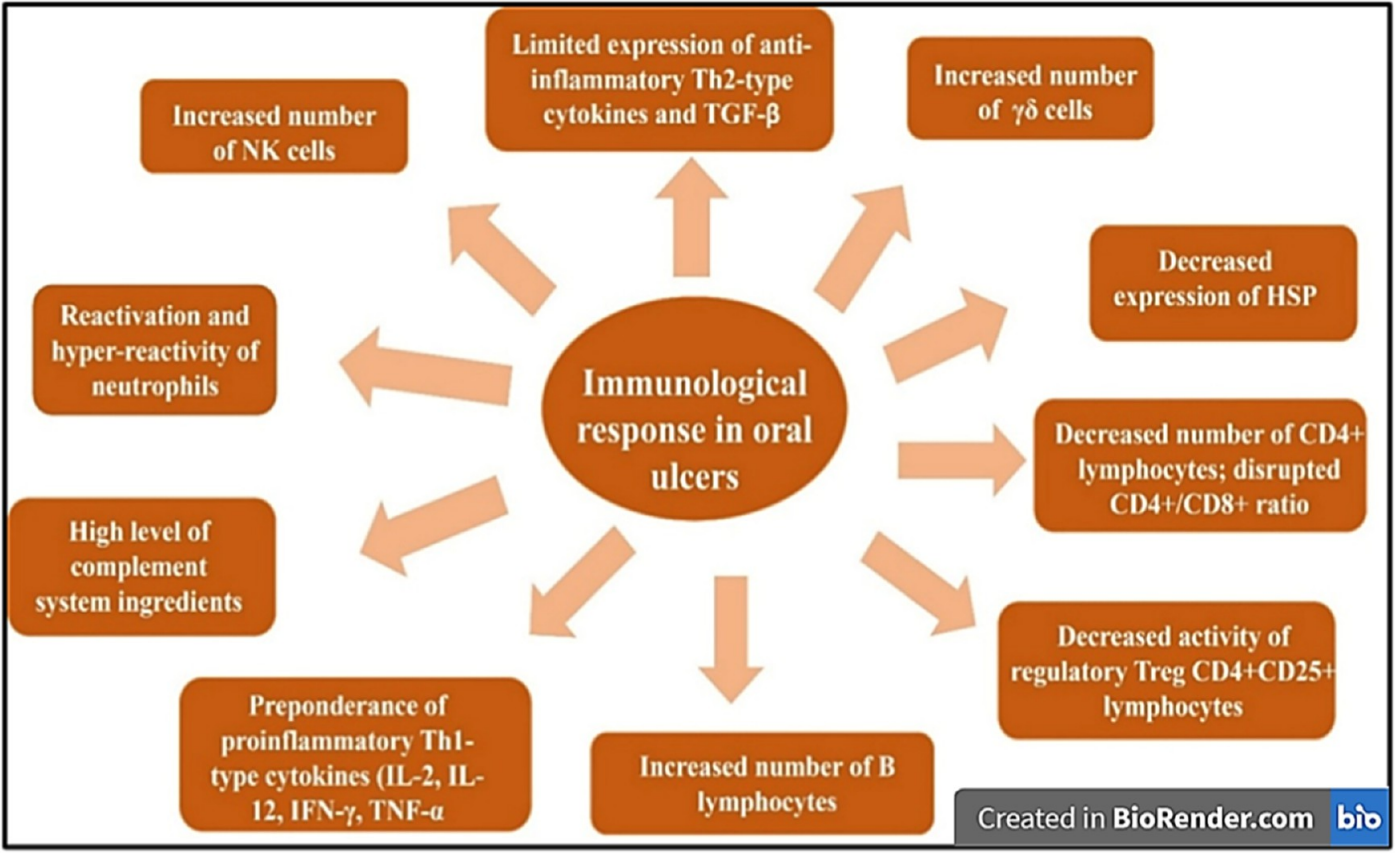
Mechanisms of the disrupted immunologic responses in oral ulcers. The schematic highlights immune dysregulation in oral ulcers, including epithelial injury–induced antigen presentation, CD8^+^ T-cell–mediated apoptosis, and cytokine imbalance (↑TNF-α, IL-2, IFN-γ; ↓anti-inflammatory signals). Aberrant neutrophil activity, oxidative stress, impaired regulatory T-cell function, and complement activation collectively disrupt mucosal integrity and delay healing (created with https://biorender.com/).

Heat shock protein 27 (HSP27), known for its anti-inflammatory properties, is found at decreased levels in the oral mucosa of individuals with oral ulcers. HSP27 inhibits the expression of proinflammatory cytokines and prevents the differentiation of monocytes into dendritic cells. Its reduced expression may exacerbate inflammatory responses, while elevated levels observed in smokers might explain the lower incidence of oral ulcers in this group.^117^ The immune dysregulation in oral ulcers encompasses both innate and adaptive responses, including hyperactive neutrophils, increased natural killer (NK) cells, and altered CD4+/CD8+ T cell ratios. These changes contribute to the pathogenesis of oral ulcers and highlight the complexity of its immunological underpinnings.^114^

Taken together, oral ulcers represent a complex clinical entity with multifactorial etiopathogenesis encompassing immunological dysregulation, genetic predisposition, and environmental triggers. Key immunopathogenic features include aberrant T-cell responses and cytokine imbalances, which contribute to mucosal breakdown and impaired healing. Despite extensive clinical and research efforts, the precise precipitating factors remain incompletely understood and the condition remains unpredictable. Genetic and immune-mediated mechanisms are increasingly recognized as central contributors, yet their management remains largely symptomatic.

Current treatment strategies are tailored to the severity, duration, and symptomatology of the lesions. Therapeutic goals are 3-fold: (a) to alleviate pain, (b) to promote mucosal healing, and (c) to prevent recurrence. Modalities range from topical agents and systemic immunomodulators to nutritional supplementation and barrier-forming formulations. Current treatments for oral ulcers are summarized in Table S1. Despite ongoing advances, oral ulcers continue to pose a clinical challenge due to their recurrent nature and multifactorial etiology. Emerging insights into immune dysfunction offer promise for more targeted and effective therapies, potentially improving the long-term outcomes for affected individuals.

## 4 Breaking Barriers with Microneedles

The history of microneedle (MN) technology development extends over 40 years.^118^ The first MN design was patented in 1976 by the United States Patent and Trademark Office, followed by the patent of a hollow MN device for intradermal drug delivery in 1996, which introduced the idea of microscale needles.^119^ Gerstel and Place drafted the patent.^120^ Microneedles (MNs) are microscopic needles, typically ranging from 10 to 1000 μm in length, designed to penetrate the outer layers of the impermeable biological substrate.^121^ The development in the microfabrication industry has accelerated the more precise and controlled production of MNs. Various types of MNs include solid, coated, hollow, dissolving, and hydrogel-based. The most recent design of MNs for skin penetration is the hydrogel-forming MN, which was fabricated and reported by Donnelly and his colleagues in 2012.^122^ Recently, researchers have devoted substantial attention to dissolving metal nanoparticles (MNPs), inventing superior materials for fabrication, developing novel designs, and optimizing scalable production techniques.

MNs have become more popular as a result of extensive research. This growing field encompasses drug delivery to various tissues (eye,^123^ buccal mucosa,^124^ gastrointestinal system,^125^ and skin^126^) and cosmetic^127^ and diagnostic uses.^128^ In 1997, a skin-perforating device was developed, and in 1998, silicon solid MNs were utilized for the first time to penetrate calcein transdermally.^129^ To inject a solution of the drug into the skin, researchers created hollow MNs in 2000.^130^ In 2004, the first coated MNs were designed to improve desmopressin transdermal administration.^131^ Drug-loaded dissolving MNs were later developed in 2006 to administer calcein and bovine serum albumin transdermally.^129,132^

In the context of oral ulcer healing, MNs are designed to penetrate the outer layers of the buccal mucosa without causing pain or significant tissue damage.^133^ MN technology presents a promising approach in overcoming key challenges in oral ulcer treatment, including inefficient drug delivery, low patient adherence, and adverse side effects, among others.^134^ Drug delivery, vaccination, and wound healing are some of the therapeutic applications for which this technology has been thoroughly studied and developed.^135^ In the treatment of oral ulcers, MN technology provides several key benefits. Its ability to penetrate the pseudomembrane barrier of oral ulcers allows for direct drug delivery to the affected site.^56^ This targeted approach minimizes drug loss and significantly enhances the therapeutic efficacy. Second, MNs are minimally invasive and virtually painless, making them a patient-friendly option, especially for individuals already experiencing discomfort from their ulcers.^133^ MN patches can be designed to release drugs in a controlled manner, ensuring a controlled therapeutic effect. This controlled release ensures that the medication is at the ulcer site for an extended period, promoting continuous healing and reducing the need for multiple applications. Combining several therapeutic agents into a single MN patch allows for combination therapy, simultaneously addressing various aspects of ulcer pathology, which can resolve the problems related to short local drug action time, low effective concentration, and single efficacy.^135^ Recent advancements in MN technology have enabled the integration of innovative formulations specifically designed for oral ulcer treatment, enhancing drug delivery efficiency and therapeutic outcomes. For instance, bioresorbable MN patches loaded with anti-inflammatory agents and growth factors have shown promising results in promoting ulcer healing and reducing inflammation.^54^ These patches adhere easily to the oral mucosa, offering a convenient and effective treatment option for patients.

MNs were first applied to the buccal mucosa by Venuganti and his colleagues in the year 2023 in a study that evaluated the feasibility of using a dissolvable MN patch to deliver 5-fluorouracil (5-FU) in an animal model for the localized treatment of oral cancer.^132^ In the study, after insertion of the MNs in phosphate buffer and excised buccal mucosa, they dissolved within 30 s and 20 min. Permeation studies in the excised porcine buccal mucosa revealed a 1.8-fold greater flux after applying the MN patch in comparison to the 5-FU solution. Later, several studies were conducted on the buccal mucosa to treat several diseases.^136^ The buccal mucosa develops a large number of microchannels as a result of the MN insertion. Characterization of various parameters, including the mechanical integrity of MNs, pain integrity, and pore closure, confirms the successful microporation of MNs in the buccal mucosa. Evaluation of the morphology, skin irritation, histological analysis, dye binding studies, cell migration assay, microchannel depth (as determined by optical coherence tomography and confocal laser scanning microscopy), and pore uniformity are the parameters for the characterization of MNs. After the insertion of MNs, the created pores gradually close due to the natural healing process and mucosal viscoelasticity.^137^

### 4.1 Polymeric Precision in Crafting MNs

The selection of the polymer plays a crucial role in the design and effectiveness of dissolving MNs for drug delivery, particularly in the application of oral ulcers. While selecting materials for the fabrication of MNs, it is necessary to ensure that their mechanical strength, dissolution rate, biocompatibility, drug-loading capacity, safety, and stability meet the clinical requirements of the FDA.^138^ Polymers used in dissolving MNs must possess adequate mechanical strength to ensure successful penetration through the biological barrier^139^ (e.g., oral mucosa) without breaking. The polymer has to be biocompatible and nontoxic to ensure safe application.^140^ To this end, natural polymers with superior biocompatibility, biodegradability, and additional wound-healing qualities, including chitosan^59^ and HA,^50^ are frequently utilized. Certain synthetic polymers, such as polyethylene glycol (PEG)-based polymers, PVA, and PVP, provide a stable environment for nanoencapsulated drugs, protecting them from degradation.^137,141^ In the case of oral ulcers, polymers with mucoadhesive properties like chitosan and gelatin can enhance localized retention, prolonging drug contact time at the ulcer site.^142^ Researchers have explored various combinations of polymers and materials for enhanced therapeutic activity.^57^ The polymers and materials used to fabricate dissolving MNs that meet the FDA requirements are depicted in Table 2.

**Table 2:** Comparative Analysis of Polymers/Composites or Materials for Dissolving MNs Used in the Treatment of Oral Ulcers^a^

polymer/composites and materials	origin	mechanical strength	biodegradable	mucoadhesive	advantages	refs
PVA	synthetic	depends on the degree of saponification	yes	no	water-soluble, excellent, biocompatible, and nontoxic	^143–145^
PVP	synthetic	high and provides structural integrity	no	no	water-soluble with good film-forming properties	^146^
PLGA	synthetic	mechanically strong	yes	yes	biocompatible, safely degrades (depending upon the MW and degree of crystallinity) and eventually disappears, and is cost-effective	^147–150^
HA	natural	moderate; suitable for oral mucosal penetration	yes	yes	biocompatible, hydrophilic, and promotes tissue hydration	^54–56^,^151^ and^152^
chitosan	natural	adequate mechanical strength in combination with other polymeric composites	yes	yes	biocompatible, hemostatic, antimicrobial, antifungal, enhanced cell proliferation and vascular regeneration, efficient drug delivery, and enhanced drug application	^59^,^145^ and^153–156^
HA and PVP	natural/synthetic	improved; PVP enhances structural integrity	HA: yes; PVP: no	yes	compatibility with hydrophilic and hydrophobic drugs, antimicrobial, and efficient drug delivery	^137^ and^141^
HA and GelMA	semisynthetic	enhanced; tunable via cross-linking	yes	yes	biocompatible, wound healing, and dual-phase drug release	^50^ and^55^
HA, PVP, and hydroxypropyl trimethyl ammonium chloride chitosan	natural/synthetic	enhanced; the combination improves mechanical properties	partially	yes	biocompatible, and mucoadhesiveness	^59^
sodium hyaluronic acid and BSP	natural	moderate	yes	yes	biocompatible, promotes wound healing, and mucoadhesive	^45^
CMCS-MA	semisynthetic	sufficient	yes	yes	biocompatible, mucoadhesive, and antibacterial properties	^53^
BSAMA and GelMA	semisynthetic	high and suitable for structural applications	yes	yes	biocompatible, tunable mechanical properties, and supports cell adhesion	^51^
ZIF-8	synthetic	good mechanical strength and puncture performance	partially	no	high drug loading capacity, and pH-responsive release	^58^
Mg-MOF	synthetic	high and suitable for structural applications	partially	no	biocompatible, pH-responsive drug release, andpromotes tissue regeneration	^57^

a PVA—poly­(vinyl) alcohol; PVP—polyvinylpyrrolidone; PLGA—poly­(lactic-*co*-glycolic acid); HA—hyaluronic acid; GelMA—gelatin methacryloyl; BSP—*Bletilla striata* polysaccharide; CMCS-MA—methacrylated carboxymethyl chitosan; BSAMA—bovine serum albumin methacryloyl; ZIF-8—zeolitic imidazolate framework-8; Mg-MOF—magnesium metal–organic framework.

### 4.2 Types of MNs

MN technology has emerged as a promising strategy to overcome the physiological and mechanical barriers associated with oral drug delivery. To address the unique challenges posed by the oral mucosa, various types of MNs have been developed, including solid, coated, dissolving, hollow, and hydrogel. These systems are fabricated using a range of materials such as silicon, stainless steel, sugar, and biodegradable polymers, each offering distinct advantages in terms of drug release mechanisms, biocompatibility, and patient comfort.^157^ Figure 8 illustrates the mechanisms of action for these types of MNs, highlighting their interaction with the oral mucosal layers. In the context of this perspective, we primarily focus on dissolving MNs, owing to their potential for painless administration, rapid drug release, and complete biodegradability for oral ulcer therapy. Detailed discussions on the design, function, and attributes of other types of MNs (hydrogel MNs, solid MNs, hollow MNs, and coated MNs) are provided in the Supporting Information file (Sections S4.2.1–S4.2.4), respectively. Consistent with the scope of this present review, our focus centers on dissolving microneedles (DMNs), highlighting their mechanistic precision in delivering biomolecules for targeted oral ulcer therapy (Section 4.3).

**Figure 8:**
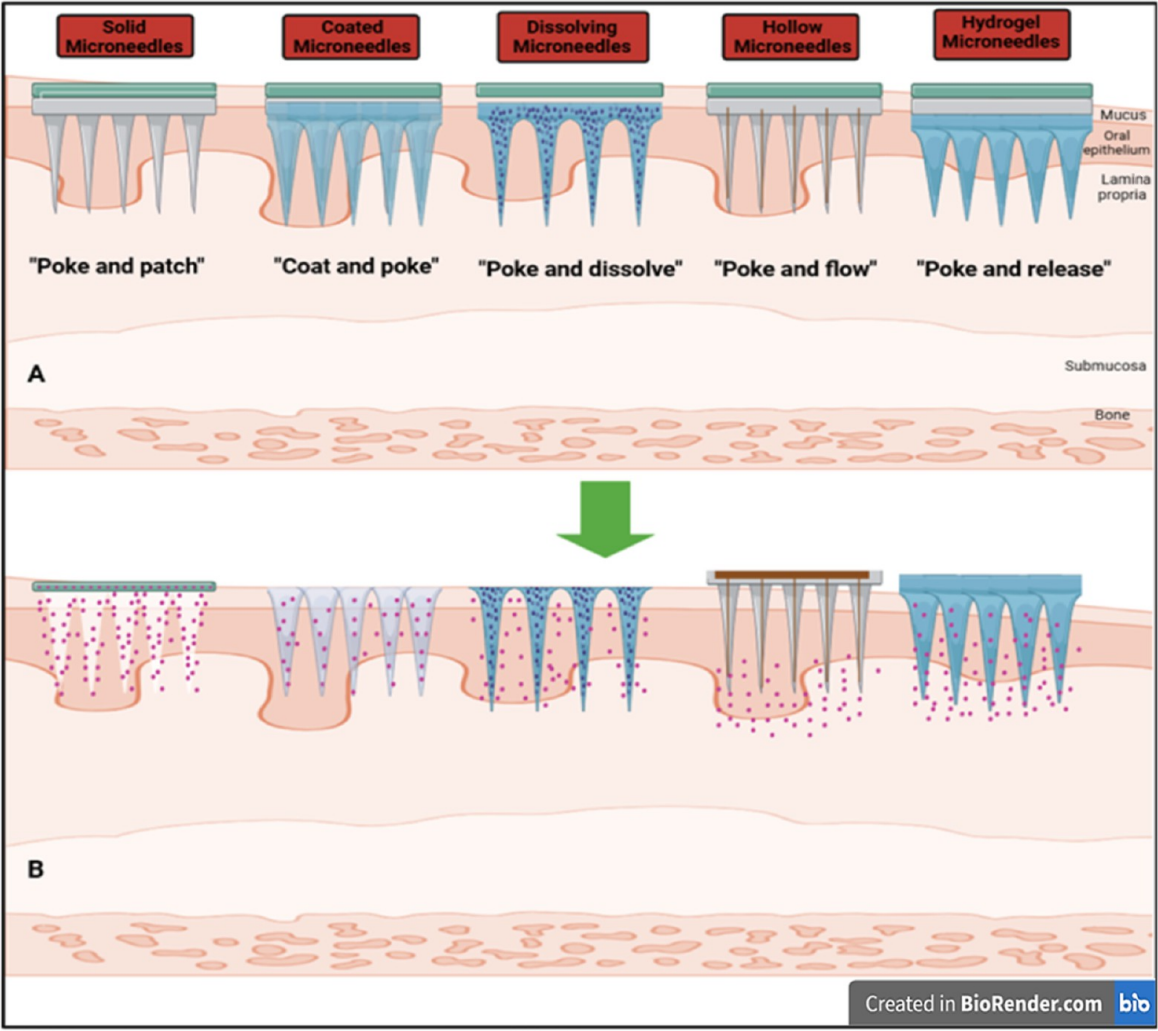
Schematic representation of different types of MNs used for drug delivery through the oral mucosa: solid, coated, dissolving, hollow, and hydrogel. Each MN type is illustrated with its mechanism of action, “poke and patch” (solid), “coat and poke” (coated), “poke and dissolve” (dissolving), “poke and flow” (hollow), and “poke and release” (hydrogel) as they penetrate the oral epithelium and underlying tissues to deliver therapeutic agents. The central panel (A) shows the native oral mucosa, while panel (B) depicts drug release (pink dots) from MNs into the lamina propria and submucosa (created with https://biorender.com/).

### 4.3 Dissolving MNs

Dissolving MNs (DMNs) was first reported in 2005 ^158^ and is a promising technique based on its advantages. Reported advantages include promoting the rapid release of macromolecules in a single-step drug application, which enables the ease of administration of the drug.^159^ After contact with the buccal mucosa, the tips dissolve and the bioactive molecule is released (poke and dissolve technique). This method is considered superior to others because of the improvement in the application of dissolvable MNs after “poke and dissolve”.^160^ A two-step casting process can quickly load the dissolvable MN tip. When the dissolvable MN is inserted into the buccal mucosa, the needle tip dissolves, releasing and diffusing the bioactive molecule rapidly.^161^ Water-soluble materials, biocompatible polymers, or sugars like hyaluronic acid (HA),^54^ poly­(lactic-*co*-glycolic acid) (PLGA),^147^ chitosan,^162^ polyvinyl pyrrolidone (PVP),^163^ poly­(vinyl) alcohol (PVA),^141,164^ dextran,^165^ carboxymethyl cellulose (CMC),^166,167^ maltose,^168^ mannitol,^169,170^ trehalose,^171,172^ and sucrose^173^ are the most appropriate materials for the manufacturing of dissolvable MNs for various therapeutic approaches. The micromold fabrication method is the most suitable for the preparation of dissolvable MNs.^174^ As the drug release kinetics of the bioactive molecule depend on the degree of dissolution of the respective polymers, it is possible to control drug delivery by adjusting the polymer composition or modifying the manufacturing process. There is a surge of interest in dissolving MNs made of biodegradable materials, as they enable the delivery of bioactive molecules without creating sharp, biocontaminated, and nondegradable waste.^175^ Moreover, manufacturing costs are significantly lower in producing MNs from semisynthetic and synthetic polymers and sugars.^176^ For long-term use, however, the primary drawback is the accumulation of polymers in the mucosa, which is undesired.^177^ Degradable MNs, a subclass of dissolving MNs, can deliver a wide range of hydrophilic drugs, including caffeine, lidocaine, metronidazole, ibuprofen, and several biopharmaceutical molecules (low molecular weight heparin, insulin, leuprolide acetate, erythropoietin, and human growth hormone).^178^ The design and fabrication of these MNs require technical expertise and are designed for complete insertion, guaranteeing optimal performance when successfully applied, and their dissolution is precisely controlled to maximize efficacy.^179^

## 5 Painless Innovation: Polymeric MN-Driven Healing for Oral Ulcers

Recent studies have explored using dissolving MN patches as an innovative approach for treating oral ulcers, offering a pain-free and efficient drug delivery system. Researchers have developed dual-functional core–shell MN patches, where the inner core, made of gelatin methyl methacrylate (GelMA), encapsulated dexamethasone, while the outer shell of the MN tips made of HA and filled with lidocaine.^142^ The combination’s HA shell was designed to dissolve quickly when applied, allowing lidocaine to be released instantly to cause analgesia at the ulcer site and lessen the accompanying discomfort. GelMA simultaneously provided mechanical strength and guaranteed dexamethasone’s continuous release. A CCK-8 assay determined the optimal dexamethasone concentration at 8 μg/mL, maximizing the cell viability. Rhodamine B-based drug release studies showed a sustained release of dexamethasone over 24 h, reducing toxicity, while lidocaine achieved a 72% release within 1 min for rapid pain relief. Biocompatibility assessments with fibroblasts (3T3) and human umbilical vein endothelial cells (HUVECs) revealed no cytotoxicity, which was further confirmed by fluorescence microscopy. ELISA tests revealed a significant reduction in inflammatory markers (IL-6, TNF-α, IL-1β), maintaining the anti-inflammatory efficacy of dexamethasone. In the SD rat oral ulcer model, MNs adhered well to the mucosa and promoted the fastest healing with the thickest granulation tissue. Systemic toxicity tests showed no weight loss or organ damage. These findings assured that DMNs are an efficient method for precise drug delivery, improving ulcer healing outcomes.

A recent study introduced a novel dissolving MN patch composed of astragalus polysaccharide (APS) and polyvinylpyrrolidone (PVP), designed to promote oral ulcer healing by inhibiting ferroptosis through the Nrf2/HO-1/SLC7A11 signaling pathway.^146^ The APS-MN patch was composed of 400 (20 × 20) uniformly shaped, pyramid-tipped needles, each measuring 700 μm in height with a 350 μm spacing, ensuring consistent geometry and effective mucosal penetration (Figure 9a–f). Mechanical testing demonstrated a maximum penetration force of 35.34 N and a compressive strength of 0.1623 MPa. Figure 9g illustrates that the insertion of APS-MN into the ventral tongue mucosa of mice resulted in observable puncture channels within the tissue, with the interchannel spacing accurately reflecting the configuration of the MN tips.

**Figure 9:**
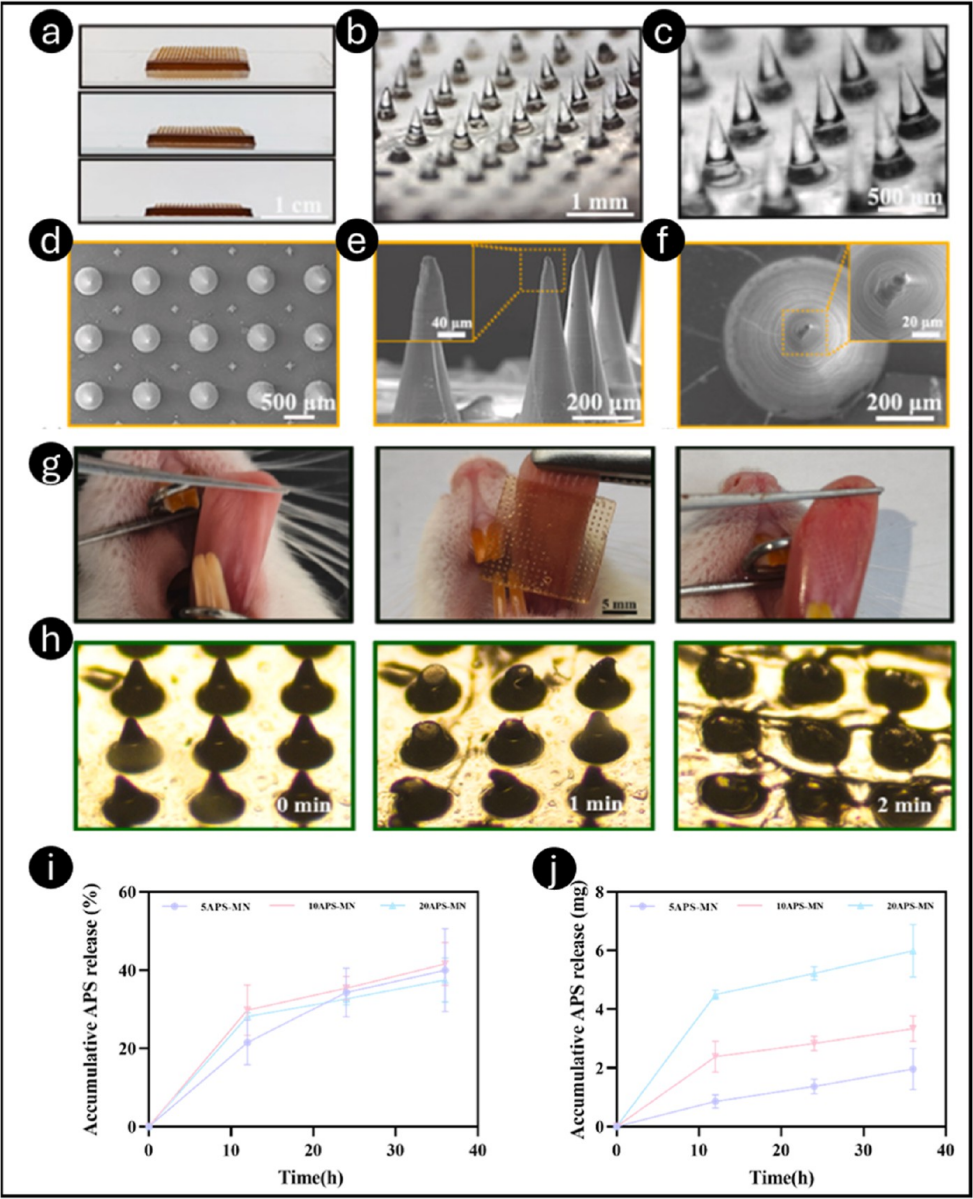
Characterization of APS-MN patches (a–c); stereomicroscopic images captured for intact and magnified APS-MN (d–f). Photographic images captured for oral mucosal penetration in rats (g). Within 2 min of penetrating the oral mucosa of rats, the APS-MN patch dissolves entirely (h). *In vitro* cumulative rate of APS (i) and cumulative APS release (j). Reprinted with permission from ref 146 Copyright 2025, Elsevier.

*In vitro* studies showed a burst release within the first 12 h, followed by a sustained release over time (Figure 9h–j). Calcein AM/PI staining revealed that both HOK and RAW264.7 cells remained predominantly viable after treatment with the APS-MN patches, indicating minimal cytotoxicity. RAW264.7 macrophages treated with APS-MNs had a lower DCFH-DA fluorescence intensity, indicating reduced intracellular ROS. *In vitro* and *in vivo* investigations revealed that APS-MN facilitated M2 macrophage polarization, diminished proinflammatory cytokines (TNF-α, IL-1β), and obstructed ferroptosis through the Nrf2/HO-1/SLC7A11 pathway, resulting in 88.2% wound closure in rats within 6 days. Notably, treatment with APS reduced lipid peroxidation and iron overload. APS also restored GPX4, an enzyme that detoxifies lipid peroxides to avoid ferroptosis. Collectively, the findings highlight promising treatment avenues for managing oxidative stress and ferroptosis-driven pathologies such as oral mucosal injury.

Wang et al. developed a multidrug dissolvable MN patch (ROUMN), designed for targeted drug release to treat oral ulcers.^56^ The HA-based MN patch combined dexamethasone acetate for anti-inflammatory effects, vitamin C for cell proliferation, and tetracaine hydrochloride for pain relief. For drug-loaded HA MN patches, 111 μL of a 1 mM dexamethasone acetate solution, 58.6 μg of vitamin C powder, and 5.5 mg of tetracaine hydrochloride powder were added to 1 mL of HA solution. Each micromold was filled with a 200 μL aliquot of base solution, yielding 8.68 μg of dexamethasone acetate, 10.54 μg of vitamin C, and 0.99 mg of tetracaine hydrochloride in each ROUMN patch. Dissolution tests showed that HA-based MNs dissolved within 10 s, significantly faster than that of gelatin/starch MNs (10 min). The results showed that at the first time point examined, the HA MNs released 3-fold more fluorescent dye transdermally (8342 au) than the gelatin/starch MNs (2491 au) and 23.6% more than the plateau value (6750 au). Moreover, the maximum drug release quantity of the HA MNs (13,917 au) was doubled that of the gelatin/starch MNs, in addition to exhibiting accelerated and enhanced drug release. *In vitro* studies using an LPS-induced inflammation model in rat aortic endothelial cells (RAEC) confirmed that ROUMN significantly suppressed IL-6 expression, exhibiting stronger anti-inflammatory effects than dexamethasone alone. CCK-8 and scratch assays demonstrated that ROUMN enhanced cell viability and migration, accelerating ulcer healing. *In vivo* studies using a rat oral ulcer model showed that ROUMN-treated ulcers healed completely within 5 days, outperforming other treatments. Histological analysis revealed early basal layer formation and full epithelial coverage by day 5, with IL-6 immunostaining confirming reduced inflammation. These findings highlighted the ROUMN patch as a promising and rapid treatment for oral ulcers, integrating anti-inflammatory, regenerative, and analgesic properties.

Bacterial infections, inflammation, and ROS make oral ulcer wounds difficult to heal. Thus, the eradication of bacteria, elimination of ROS, and mitigation of inflammation are essential for the management of oral ulcerations. Oligomeric proanthocyanidins (OPC) and 3-(aminomethyl) phenylboronic acid-modified hyaluronic acid (HP) formed polymer gels through dynamic covalent borate bonding was reported in the study by Zhang et al.^180^ Minocycline hydrochloride (MH) was injected into the polymer gel, and vacuum-prepared multifunctional MH/OPC-HP-MNs with ROS-responsive characteristics were created (Figure 10a–f). OPC and MH loadings in MNs were 164 and 6.3 μg, respectively. MH/OPC-HP gel MNs in PBS released the OPC more slowly than MH alone, which released 65.0% cumulatively at 24 h. In contrast, incubating MH/OPC-HP MNs in 0.1 mM H_2_O_2_ boosted OPC release by 79.2% at 24 h, indicating significant H_2_O_2_ sensitivity. The rapid HA disintegration caused the MH/HA MNs to release 83% of MH in PBS within 30 min. MH release from cross-linked MH/OPC-HP gel MNs in PBS was slower, reaching 80% cumulative release after 2 h (Figure 10g,h). Gel-based MH/OPC-HP MNs prolonged oral ulcer OPC retention and ROS scavenging. MH/OPC-HP MNs were biocompatible in cytocompatibility and hemocompatibility tests. Antibacterial tests showed that MH-loaded MNs were effective. *In vitro* tests showed that MH/OPC-HP MNs cleared ROS, reduced oxidative stress damage, inhibited M1-type macrophage polarization, and induced M2-type. *In vivo* tests showed that MH/OPC-HP MNs inhibited proinflammatory cytokines, promoted neovascularization, accelerated ulcer epithelial repair, and dramatically improved oral ulcer wound infection in rats. In conclusion, MH/OPC-HP MNs may improve the oral ulcer wound healing.

**Figure 10:**
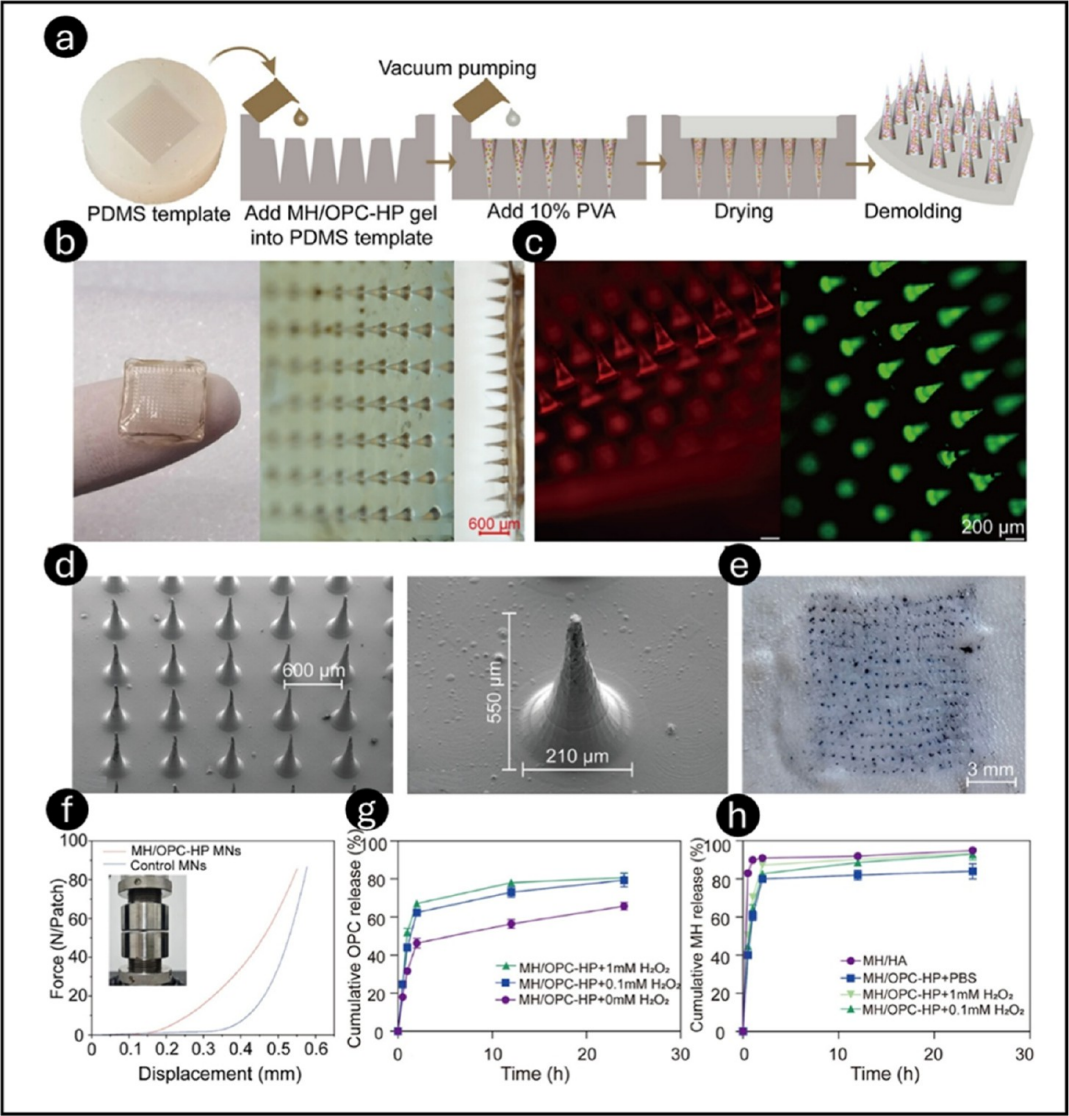
Characterization of MH/OPC-HP MNs. Methods of preparation of MH/OPC-HP MNs (a). Photograph of MH/OPC-HP MNs, scale bar: 600 μm (b). Fluorescence images of MH/OPC-HP MNs, scale bar: 200 μm (c). SEM images of MH/OPC-HP MNs (d). Representative images of rat skin following the application of MH/OPC-HP MNs, scale bar: 3 mm (e). Mechanical properties testing of MH/OPC-HP MNs (f). Cumulative *in vitro* release of OPC (g) and MH (h) from the MNs system at different concentrations of H_2_O_2_. All data are presented as mean ± SD (*n* = 3). Reprinted with permission from ref 180 Copyright 2025, American Chemical Society.

Zheng et al. developed a multifunctional MN patch using HA and hydroxypropyl trimethylammonium chloride chitosan (HACC) loaded with dexamethasone (DXMS) and basic fibroblast growth factor (bFGF) for oral ulcer healing.^59^ This structural design enabled the effective penetration of the oral mucosal tissue. The patch exhibited strong mechanical properties, penetrated the buccal mucosa to a depth of 400 μm, dissolved within 2 min, and efficiently delivered drugs to deep ulcer sites. *In vitro* studies confirmed excellent biocompatibility, with bFGF promoting cell proliferation, migration, and neovascularization, while DXMS provided anti-inflammatory effects by reducing TNF-α and IL-6 levels in HOrF and HUVEC. These findings confirmed the excellent biosafety of the MN patches and their potential to promote cell activity and proliferation, highlighting their promising application in oral ulcer treatment. HACC enhanced antimicrobial activity against *E. coli, S. aureus, S. mutans,*and*C. albicans* without interfering with drug function. Scratch and tube formation assays demonstrated improved cell migration and capillary formation, accelerating tissue repair. *In vivo* studies using an SD rat model showed that HA/HACC@DXMS&bFGF MN patches achieved complete ulcer closure by day six, outperforming other treatments. Macrophage polarization analysis indicated effective immunomodulation, and histopathological examination confirmed biosafety. These findings highlighted the MN patch’s potential as an effective oral ulcer treatment by combining anti-inflammatory, proangiogenic, and tissue-regenerative properties.

In another study by Guo et al., a soluble HA MN patch (BSP-BDP@HAMN) with betamethasone 17,21-dipropionate (BDP) and betamethasone 21-phosphate sodium (BSP) was created to treat oral ulcers.^55^ The needle body of MNs comprised 248 μg of BSP and 620 μg of BDP. Furthermore, the composite film comprised 540 μg of BSP and 1.35 mg of BDP. BSPBDP@HAMNs had sufficient mechanical strength to penetrate the rat tongue’s mucosa, with an insertion depth of 207 ± 3 μm. BSP and BDP were released into the ulcer base by the swiftly dissolved HA MN carrier within 3 min of mucosal penetration (Figure 11). BSP-BDP@HAMNs showed high biocompatibility, with primary hGFs retaining morphology and viability above 80% at 50–300 μg/mL concentrations. MNs increased cell migration and proliferation, as shown by scratch and CCK-8 experiments. In LPS-induced inflammation models, BSP-BDP@HAMNs dramatically lowered TNF-α levels, demonstrating their potent anti-inflammatory effects. *In vivo* studies demonstrated effective MN insertion into rat tongue mucosa, delivering drugs to depths of 207 ± 3 μm. The MNs disintegrated within 3 min, releasing the therapeutic molecules quickly. BSP-BDP@HAMNs was faster at healing oral ulcers than other therapies. By day 5, the ulcer healing rate was 87.4%, surpassing the triamcinolone dental paste-treated group (79.1%) and control group (49%). Histological investigation showed that BSP-BDP@HAMNs healed tissue, reduced inflammation, and enhanced collagen and neovascularization. Immunohistochemical experiments demonstrated strong TNF-α suppression, indicating anti-inflammatory and prohealing effects. These data suggested BSP-BDP@HAMNs for effective oral ulcer treatment.

**Figure 11:**
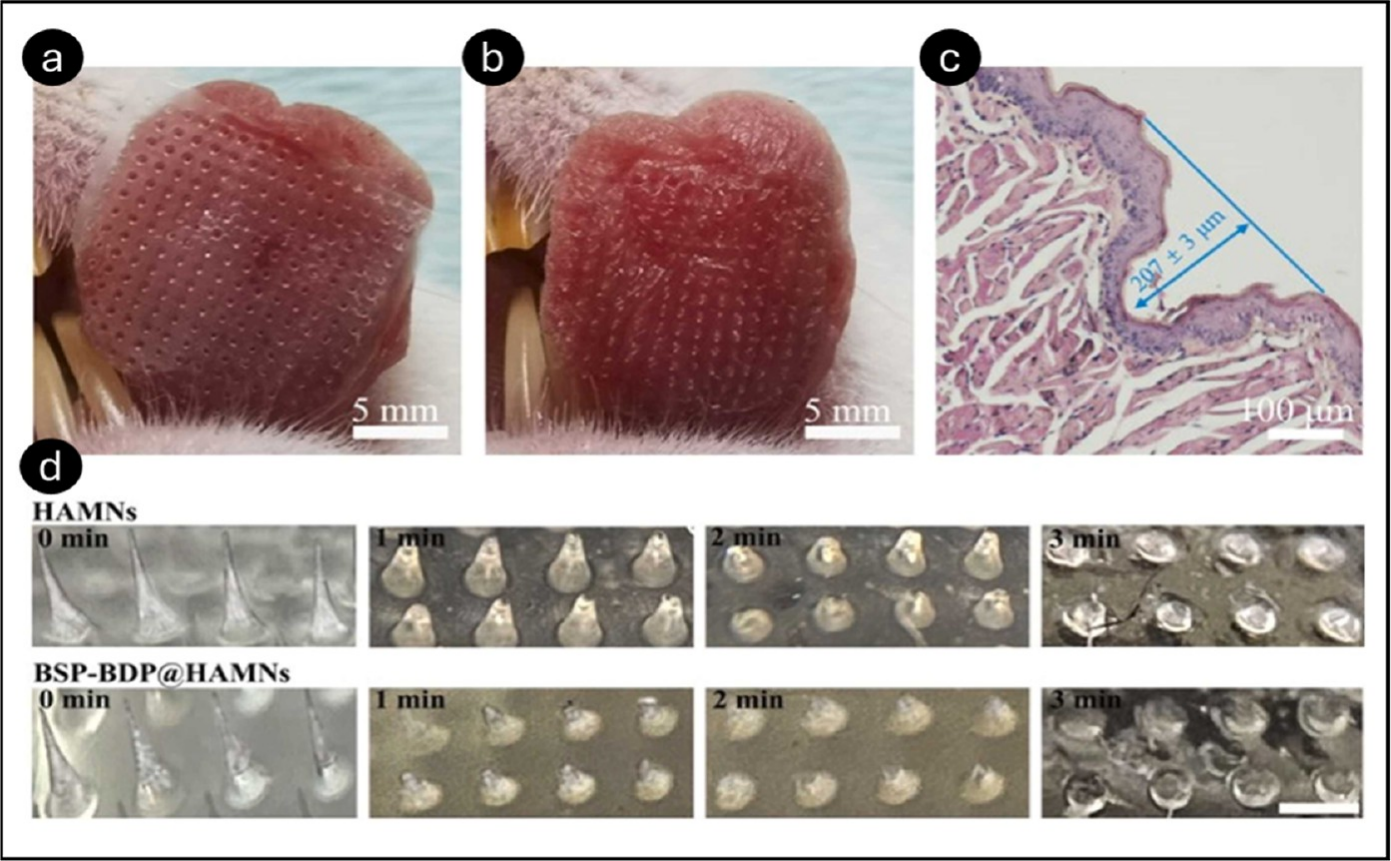
A comparison of captured images between the rat tongue tissue before (a) and after (b) the introduction of BSP-BDP@HAMNs. Rat tongue tissue stained with H&E after being exposed to BSP-BDP@HAMNs (c). The dissolution procedure of the HAMNs and BSP-BDP@HAMNs with respect to time (d). The bar represents the scale, which is 0.5 mm. Reprinted with permission from ref 55 Copyright 2023, Elsevier.

DMN loaded with HA-PVP and betamethasone 21-phosphate sodium (BSP) was developed by Li et al. to painlessly penetrate the oral mucosa barrier and deliver drugs directly to the submucosa or basal layer.^141^ The drug loading of BSP in HA-PVP MNs was quantified by the drug quantity in a single array of 100 MNs (10 × 10), as assessed by HPLC. The quantity of BSP loaded in a single array of HA-PVP MNs exhibited a linear correlation with the drug concentration in the HA solution. At a BSP concentration of 70 mg/mL in HA solution, the drug loading of HA-PVP MNs was around 0.48 mg, deemed appropriate for clinical dosage. Biocompatibility of HA-PVP MNs was evaluated using the CCK-8 assay on HOK cells. The results showed no cytotoxicity, and at concentrations of 1 mg/mL and above, HA-PVP MNs even promoted cell proliferation due to their water-retaining and viscoelastic properties. A protective backing layer was added to the MN patch to address drug loss from saliva flow. The MN patch exhibited strong adhesion to oral mucosa and remained attached for over 3 h in artificial saliva. Saliva flow-through studies showed that HA-PVP MNs dissolved rapidly (<1 min) without the backing layer, leading to drug loss (Figure 12a). However, the backing layer retained the drug and diffused it into the mucosa through the MN pores. The EC waterproof layer could then be removed, ensuring efficient drug delivery. The study evaluated the mucosal penetration, recovery, and biosafety of the MN patch in rats. Fluorescence imaging demonstrated that the MN patch significantly enhanced drug penetration, with a strong FITC-BSP signal in the basal layer within 60 min, which is much higher than that of a topical drug solution. MNs were partially dissolved upon penetration, aiding drug release. Mucosal irritation studies showed minimal tissue damage. While pinholes were visible immediately after MN application, they disappeared within 2 h, and the mucosa fully recovered in 12 h, with no signs of erythema or edema in male SD rats (Figure 12b). These findings confirmed the MN patch’s biosafety, making it a promising, efficient, and painless drug delivery system for oral mucosal diseases.

**Figure 12:**
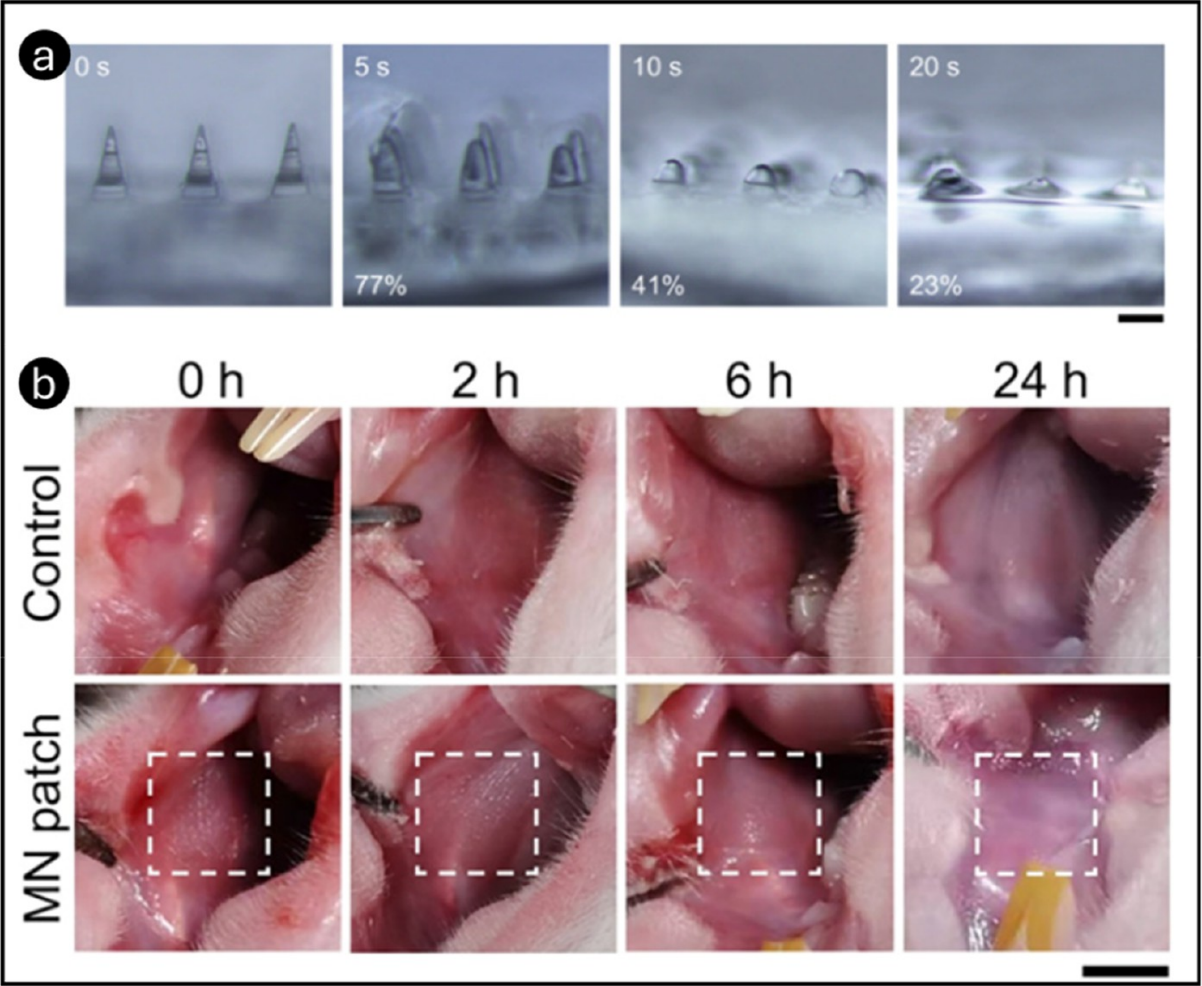
Microscopic photos of HA-PVP MNs penetrated and retained in rabbit oral mucosa for varying durations (0–20s). Scale bar = 200 μm (a). Images depicting the recuperation of rat oral mucosa following MN patch insertion (scale bar = 2 mm) (b). Reprinted with permission from ref 141 Copyright 2022, Elsevier.

Another study by Yu et al. underscored the development of a new method for treating oral ulcers, a DMN patch with a core–shell that contains numerous medications. A HA/gelatin methacryloyl core–shell MN patch was designed for the comprehensive treatment of oral mucosal ulcers.^50^ The MNs were composed of a basic fibroblast growth factor (bFGF) methacrylate gelatin shell, a dexamethasone (DXMS)-loaded hyaluronic acid (HA) core, and zeolite imidazoline framework-8 (ZIF-8) encased in the HA-based backplane. The gradual breakdown of gelatin methacryloyl (GelMA) at the MN patch’s tip in the oral mucosa led to a steady release of bFGF at the lesion site, which greatly enhanced cell migration, proliferation, and angiogenesis. Due to the rapid disintegration of the core section of HA, practically all DXMS and ZIF-8 were released from MNs within 3 and 4 min, respectively. After delayed GelMA disintegration for >7 days, the shell structure released bFGF constantly. These results indicate that the core–shell MN patch can release drugs programmatically (Figure 13a–d).

**Figure 13:**
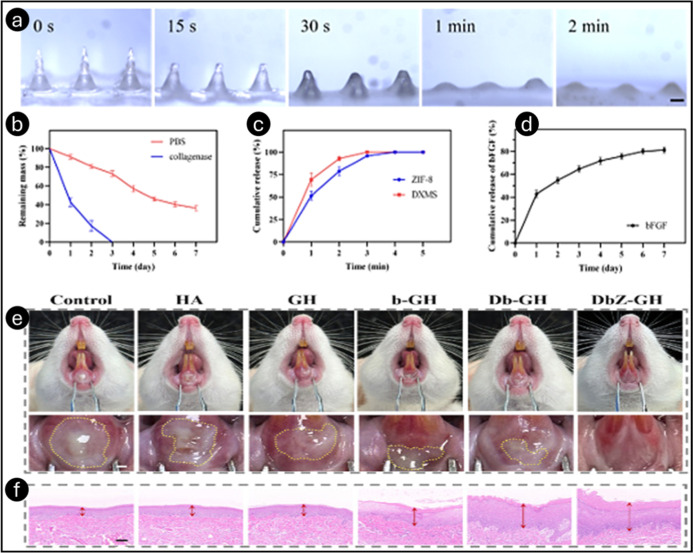
Microscopic images depicting the dissolving characteristics of GelMA@bFGF/HA@DXMS&ZIF-8 (DbZ-GH) MNs following various durations of insertion into the rat oral mucosa (a). *In vitro* degradation patterns of the DbZ-GH MN patches immersed in phosphate-buffered saline and collagenase (b), respectively. The release profiles of DXMS and ZIF-8 from DbZ-GH MNs (c). *In vitro* cumulative release of bFGF from the DbZ-GH microneedle patch (d). All data are presented as mean + SD (*n* = 3). *In vivo* utilization of MNs for the treatment of oral mucosal ulcers. Overall pictures of ulcers on day 5 postintervention across several groups (e). Images of ulcerated tissues stained with hematoxylin and eosin, treated with different formulations (f). Reprinted with permission from ref 50 Copyright 2024, Elsevier.

MNs, loaded with 1 μg/mL bFGF, significantly enhanced cell migration and tubule formation in human oral fibroblasts (HOrF) and human umbilical vein endothelial cells (HUVEC). In addition, the anti-inflammatory benefits of the fast-released HA and DXMS were enhanced and the antibacterial properties of the remaining MN backing served as a dressing once the tip dissolved. ZIF-8 effectively inhibited *S. aureus* and *E. coli* growth, with the strongest antimicrobial effect observed at 3 mg/mL in an antimicrobial study. Live/dead staining confirmed that ZIF-8 at 3 mg/mL had excellent bacterial lethality while maintaining good biocompatibility in fibroblasts. Histological analysis revealed improved epithelial thickness and reduced inflammatory cell infiltration in the drug-loaded MN group (Figure 13e,f). MNs significantly reduced TNF-α and IL-6 levels, indicating effective inflammatory suppression. These findings highlighted dissolving MNs as a promising alternative to conventional therapies, offering precise drug delivery, rapid healing, and minimal side effects.

Ge et al. developed a multifunctional, soluble HA-MN patch to accelerate oral ulcer healing by combining anti-inflammatory, angiogenic, and antibacterial properties.^54^ The MN tip was loaded with triamcinolone acetonide (TA) and epidermal growth factor (EGF) (TEZ-HA) to reduce inflammation and promote neovascularization. At the same time, the base contained zeolitic imidazolate framework-8 (ZIF-8) for controlled Zn^2+^ release, enhancing the antibacterial activity. *In vitro* studies confirmed enhanced cell migration, tube formation, and antibacterial effects against *E. coli* and *S. aureus*, with ZIF-8 maintaining biocompatibility at 3 mg/mL. The results of the enzyme-linked immunosorbent assay demonstrated that the multifunctional HA-MN patch effectively reduced the expression levels of TNF-α and IL-6 in macrophages induced by lipopolysaccharide (200 ng/mL). This was confirmed by the fact that TA-loaded HA microneedles (T-HA) and TEZ-HA effectively reduced these levels. *In vivo* wound healing studies showed that by day 6, the oral ulcers in the TEZ-HA group had predominantly healed, exhibiting brilliant red mucosa, while the control group displayed slower-healing ulcers. H&E staining demonstrated minimal epithelialization and loosely structured connective tissue in the control ulcers, accompanied by diffuse lymphocytes, neutrophils, and macrophages in the submucosa. Conversely, the TEZ-HA group exhibited superior healing of the oral mucosa, evidenced by a 67% enhancement in submucosal epidermal thickness relative to the control group, signifying a favorable healing effect (Figure 14). Macrophage polarization analysis indicated an increased M2/M1 ratio, promoting tissue repair, and immunofluorescence staining revealed enhanced neovascularization. Finally, by presenting a new method of drug delivery, this study offered a potential approach to hastening oral ulcers.

**Figure 14:**
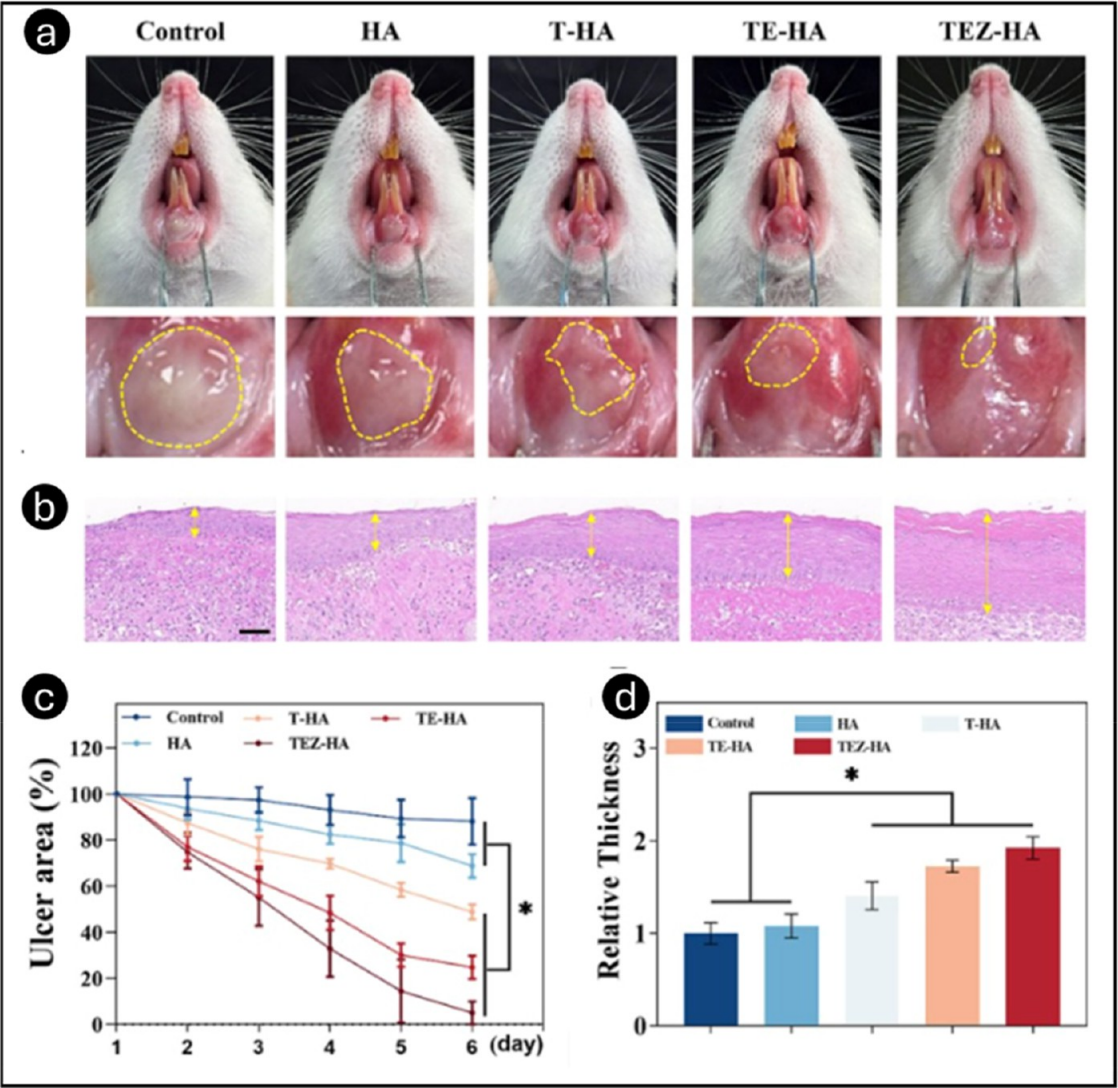
*In vivo* healing of mouth ulcers. Photographs of ulcers in rats subjected to different therapies on day six (a). Hematoxylin and eosin staining of ulcerative tissue on day six (b). Normalized quantitative assessment of the ulcer area from days 1 to 6 (c). Normalized quantitative assessment of epithelial thickness in ulcerated tissue on day 6 (d). Scale bars represent 100 μm. *P < 0.05. Reprinted with permission from ref 54 Copyright 2023, Elsevier.

Yin et al. developed dissolvable layered MNs composed of hyaluronate, cetyl pyridinium chloride (CPC), and recombinant basic fibroblast growth factor (rbFGF) to enhance the treatment of oral ulcers.^151^ A solution containing 0.4 mg of rbFGF, 2.5 mg of CPC, and 5 mL of HA was formulated. The ultimate concentrations of rbFGF and CPC were 0.08 and 0.5 mg/mL, respectively. The swift disintegration capability showed efficient delivery of the encapsulated drug to the ulcer location and mitigated the sense of a foreign body. The height and weight of the HA MN patch decreased by over 75% following a 5 min insertion into the oral mucosa, demonstrating the rapid dissolution property of the produced MNs. The MNs enabled targeted drug delivery, promoting rapid healing through rbFGF-induced cell proliferation and migration, and CPC provided antimicrobial effects. Antibacterial studies demonstrated a concentration-dependent increase in efficacy against *E. coli, S. aureus,*and*S. mutans*, with the 2.5 mg/mL formulation achieving over 99% inhibition. Biocompatibility assessments using MTT assays and Calcein-AM/PI staining against L929 mouse fibroblast cells confirmed high cell viability, with the rbFGF/CPC HA MN group showing superior fibroblast proliferation. *In vivo* studies on SD rats demonstrated significantly accelerated ulcer healing, with the rbFGF/CPC HA MN group achieving complete wound closure by day seven. These results underscore the superior therapeutic potential of the rbFGF/CPC-HA microneedle formulation in accelerating and enhancing oral ulcer healing.

Taken together, the use of polymeric dissolvable MNs for oral ulcers represents a breakthrough in painless and efficient healing. These preclinical case studies, including *in vitro* and *in vivo* experiments, demonstrate their ability to enhance therapeutic delivery, accelerate tissue regeneration, and improve patient comfort. From dissolvable MNs ensuring localized therapeutic action to bioresponsive designs enabling sustained release, these advancements highlight their superiority over conventional treatments. As research advances, polymeric dissolvable MNs continue to redefine oral ulcer therapy, promising a minimally invasive and efficient solution for accelerated healing and improved clinical outcomes, establishing them as a groundbreaking advancement in oral care. With ongoing innovations in biocompatible materials and therapeutic formulations, the future of MN-driven healing looks promising. Expanding preclinical research suggests that polymeric MNs are poised to become the preferred treatment modality for oral ulcers, ensuring better patient adherence and enhanced therapeutic efficacy. By bridging the gap between scientific advancements and real-world applications, polymeric dissolvable MNs stand as a transformative approach to modern oral healthcare.

## 6 Nanotherapeutics-Infused Dissolving MNs for Oral Ulcers

Oral ulcers, painful mucosal lesions caused by trauma, infections, or immune factors, are poorly managed by conventional therapies due to rapid salivary clearance and low drug retention.^39^ Nanotherapeutics-loaded MNs offer a breakthrough solution by enabling targeted, sustained drug delivery directly to the ulcer site, enhancing therapeutic efficacy, and healing. They offer precise penetration into the mucosal layer, bypassing the saliva barrier and directly depositing therapeutic agents at the ulcer site.^133^ By incorporating drug-loaded nanoparticles (e.g., polymeric NPs, lipid-based carriers, or exosomes) into dissolvable MNs, controlled and prolonged drug release can be achieved.^45,51,58,59^ Additionally, stimuli-responsive nanocarriers can be designed to release payloads in response to ulcer-specific conditions, such as low pH or elevated inflammatory enzymes, further enhancing treatment efficacy.^181^ Beyond improved drug delivery, they promote tissue regeneration by delivering growth factors or bioactive molecules that accelerate wound healing, while minimizing systemic exposure. Their minimally invasive nature ensures patient compliance, making them a superior alternative to conventional formulations. This subsection explores recent nanotherapeutic-integrated MN approaches developed to revolutionize oral ulcer management.

A novel dissolving MN patch integrating bone marrow mesenchymal stem cell-derived exosomes (Exos) and folic acid-magnetic nanoparticles (FMNs) within a methacrylate carboxymethyl chitosan (CMCSMA) structure demonstrated a synergistic therapeutic effect for treating oral mucosal lesions.^182^ This combination enhanced immune regulation, angiogenesis, and epithelial repair, leading to accelerated healing and pain relief. To create the MMP, 2% CMCSMA with 0.25% photoinitiator, 200 μg/mL Exos, and 10 μg/mL Fmns were cast on negative MN molds. To support the top, a 10% gelatin solution with 2% lidocaine hydrochloride was applied equally (Figure 15a–d). Developed MN patches showed a penetration depth of ∼450 μm, completely dissolved within 10 min, and provided a rapid drug release. The gelatin support layer dissolved at 37 °C in artificial saliva, releasing lidocaine linearly and dissolving within 10 min. Release of Fmns and Exos from cross-linked CMCSMA significantly altered MMP biological activity. Ion from Fmns had a burst release, followed by a steady release. Free heavier Fmns introduced into the network through noninclusion interactions may cause the burst phase. The gradual and persistent release of negatively charged Exos from MMP was caused by dissociation from positively charged CMCSMA main chains and diffusion processes, unlike Fmns (Figure 15e–g).

**Figure 15:**
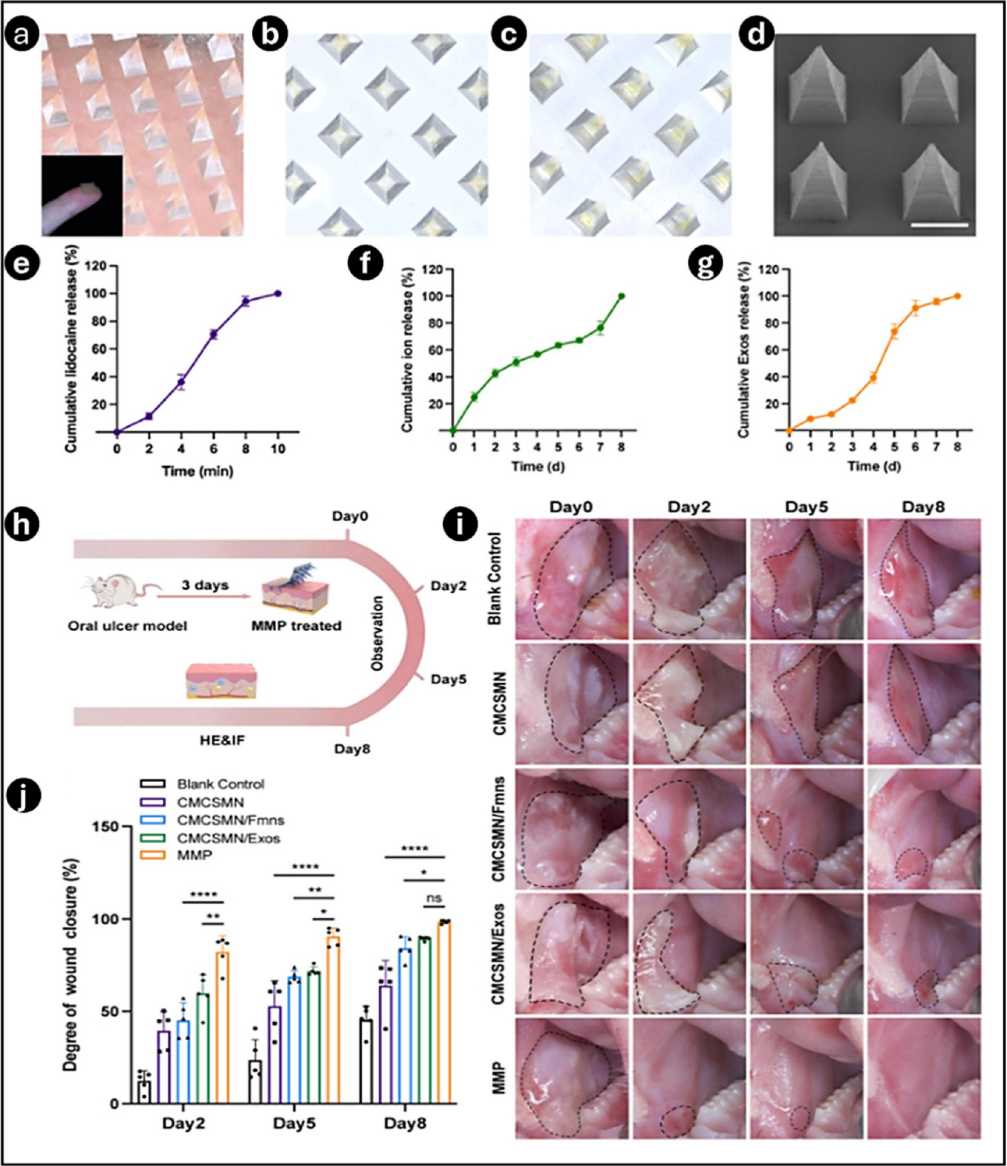
Physical MMP characterization. Digital snapshot of MN arrays and gross examination of finger-mounted MMP (a). Top and side stereomicroscopic pictures of MMP (b,c). Representative SEM images captured (d). Scale bar: 400 μm. The cumulative *in vitro* release of lidocaine, ions, and Exos was evaluated based on the swelling and disintegration of MMP in PBS at 37 °C and pH 7.4 (e–g). *In vivo* regeneration effects of MMP for oral ulcers. Schematic procedure to evaluate the therapeutic efficacy of MN patches for oral ulcers in rats (h). Gross observation of the oral ulcer healing process in SD rats treated with CMCSMN, CMCSMN/Fmns, CMCSMN/Exos, and MMP and no treatment on days 0, 2, 5, and 8 (i). Calculations of rat oral ulcer healing area results for different treatments at days 0, 2, 5, and 8 (j). n.s. indicated not significant, *p < 0.05, **p < 0.01, ***p < 0.001, and ****p < 0.0001. Reprinted with permission from ref 182 Copyright 2024, Applied Materials Today.

Antimicrobial tests against *E. coli* and *C. albicans* revealed significant bacterial and fungal inhibition, reducing colony-forming units within 24 h. *In vitro* assays showed accelerated HOK migration with MN-treated cells forming a monolayer within 6 h and complete wound closure by 12 h. *In vivo* studies in the SD rat buccal ulcer model demonstrated that MN-treated ulcers had the fastest closure rate (82.11% at day 2) and shortest healing time (Figure 15h–j). These findings highlighted the synergistic approach of exosomes in dissolving MNs, leading to enhanced wound healing, reduced inflammation, antimicrobial effects, and improved immunomodulation, making them a promising therapeutic approach for oral mucosal diseases.

A study on silk fibroin (SF)-MNs-loaded with lipopolysaccharide (LPS)-pretreated bone marrow mesenchymal stem cell-derived exosomes (LPS-pre-Exos) demonstrated the synergistic therapeutic advantages of integrating nanotherapeutics for oral ulcer management (Figure 16a).^58^ To make the SF MNs, 0.7 g of SF was mixed with 1 mL of PBS and 5 μL of lithium acylphosphonate salt (LAP 0.05%, g/mL) as a photoinitiator. The next step was the individual addition of 100 ng/mL LPS-pre-Exos and 3 mg/mL ZIF-8. Lastly, the MN backplate was made using the SF solution that had 3 mg/mL of ZIF-8. As shown in Figure 16b,c, EZ-MN’s persistent Zn^2+^ release supported sustained antibacterial action. Infiltrating MNs in PBS for 7 days allowed the gradual release of the kinetics of exosomes. After the burst release, exosomes were released continuously. The SF-MN patches demonstrated excellent biocompatibility in human buccal mucosa fibroblasts (HBMFs) and HUVECs, ensuring safety in both *in vitro* and *in vivo* models, as they did not affect cell proliferation, hemolysis, or major organ health. LPS-pre-Exos-loaded patches significantly enhanced endothelial cell migration, tube formation, neovascularization, and tissue regeneration. Their anti-inflammatory effects were also notable, as they effectively reduced TNF-α and IL-6 levels, facilitating the transition of M1 macrophages (proinflammatory) to M2 macrophages (prorepair), which supports wound healing.

**Figure 16:**
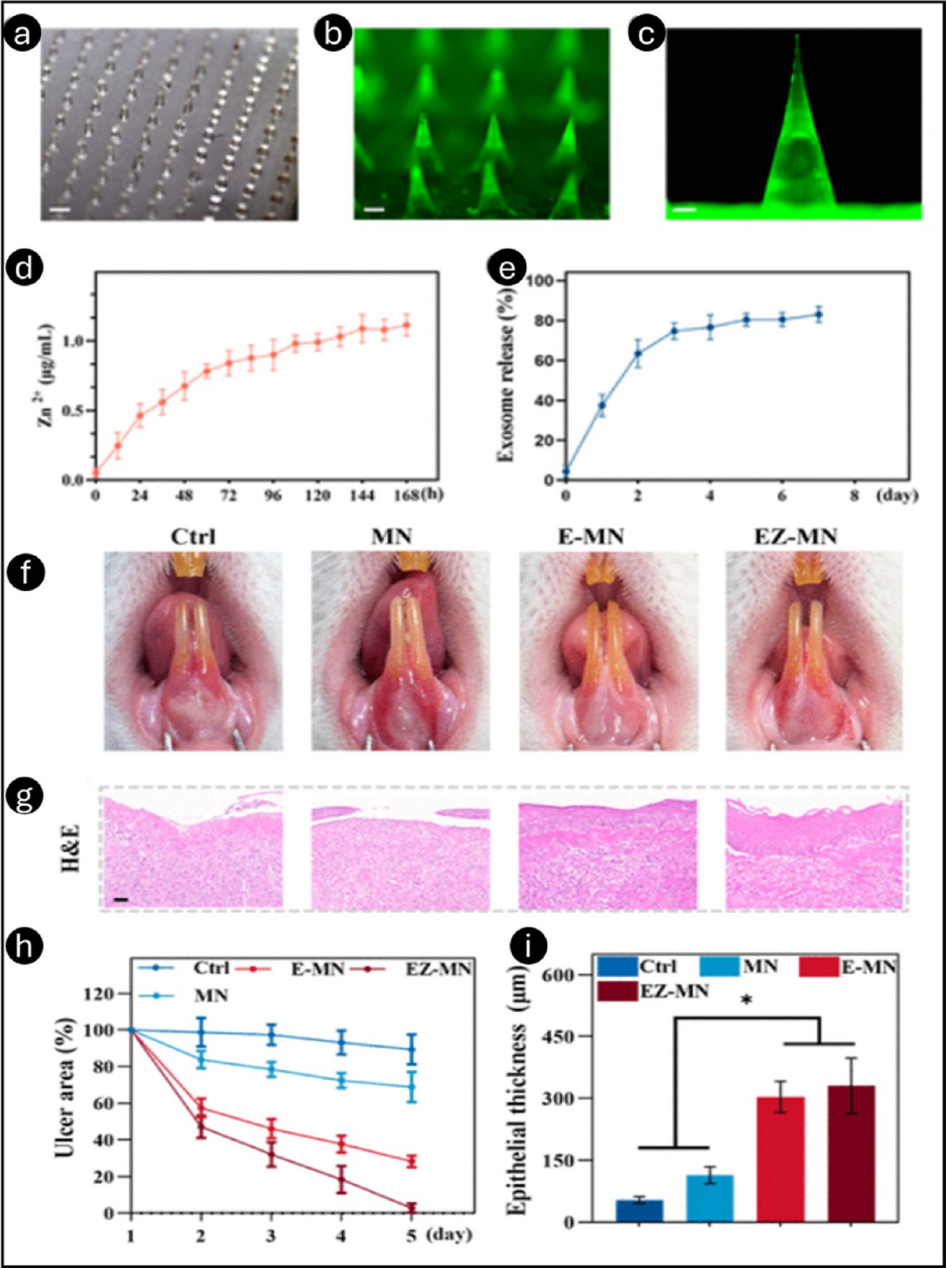
Light microscopy images of an SF MN patch (a). MNs infused with a green-fluorescent compound (b,c). *In vitro* drug release of the microneedle patches. Cumulative release of Zn^2+^ from EZ-MN (d). Cumulative exosome release from EZ-MN in PBS during a duration of 7 days (e). *In vivo* healing of oral ulcers. Photographic images showing ulcers in rats (f). The tissue surrounding an ulcer stained with H&E (g). Area of ulcer healing standardized quantitatively (h). Evaluation of the thickness of the mucosa quantitatively (i). *P < 0.05. The scale bar is 50 μm. Reprinted with permission from ref 58 Copyright 2024, American Chemical Society.

Additionally, the patches exhibited strong antibacterial properties due to the incorporation of zeolitic imidazolate framework-8 (ZIF-8) nanoparticles, effectively inhibiting the growth of *S. aureus*, *S. mutans*, and *E. coli*. *In vivo* studies in a rat oral ulcer model demonstrated that patches significantly accelerated wound healing (Figure 16f–i). By day 5, ulcers in the MN-treated group were nearly healed, whereas the control group showed a delayed recovery. Overall, the findings revealed the significance of integrating LPS-pre-Exos and ZIF-8 nanoparticles into dissolving MNs, therefore providing a multifunctional approach to oral ulcer treatment by promoting rapid healing, reducing inflammation, and preventing bacterial infections.

Recently, the development of magnesium metal–organic framework (Mg-MOF)-MNs loaded with curcumin (CUR) offered a synergistic strategy for accelerating oral ulcer healing.^57^ Briefly, a hydrothermal synthesis approach was used to produce Mg-MOF (Figure 17a–c). After ultrasonically treating CUR in ethanol, it was combined and agitated with Mg-MOF for 24 h. HA MNs patches were utilized to encapsulate MC and EPL, hence enhancing the efficiency and effectiveness of drug administration for ulcer healing, resulting in HA@EPL&Mg-MOF@CUR (HEMC) MNs patches (Figure 17d–f). The release rates of Mg-MOF and CUR from the HEMC MNs are shown in Figure 17g,h. A controlled-release profile was noted for both Mg-MOF and CUR. Compared to other Mg-MOF-based therapeutic strategies, the engineered HEMC-MNs exhibited sustained drug release kinetics and superior release efficiency. The synergistic action of Mg-MOF and CUR enhanced the antioxidant capacity, outperforming previously used CUR-loaded polymeric formulations. Notably, incorporation of MC and EPL into HEMC-MNs enhanced their antibacterial properties. MNs significantly reduced inflammation by downregulating proinflammatory cytokines TNF-α and IL-6. The immunomodulatory effects were evident in macrophage polarization, where treatment with MNs shifted M1 macrophages (pro-inflammatory) toward M2 macrophages (pro-reparative), supporting tissue repair.

**Figure 17:**
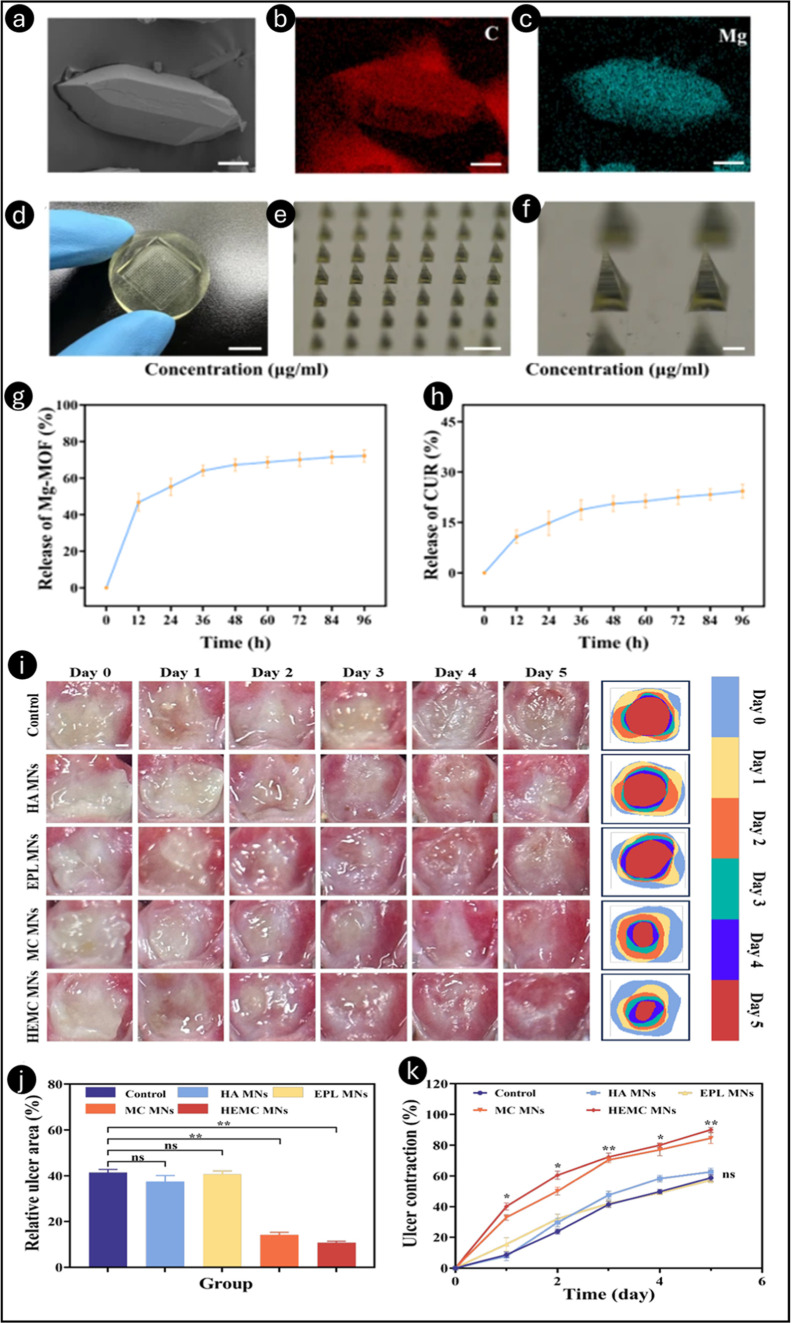
SEM characterization of MG-MOF and MC (a–c). Scale bars: 1 μm. Digital photos of PDMS MNs molds (d). Light microscopy images of HEMC MNs (e,f). *In vitro* release profile of Mg-MOF (g) and CUR (h) in HEMC MNs. *In vivo* evaluation of ulcer healing in rats with mouth ulcers. Digital photos depicting ulcer healing in rats after therapy across various MN groups, accompanied by a simulation of the ulcer healing process (i). Scale bar: 1 mm. Quantitative assessment of the area of persisting unhealed ulcers at the time of rat euthanasia (j) and quantitative investigation of daily ulcer healing (k). Reprinted with permission from ref 57 Copyright 2024, Springer Nature.

*In vitro* and *in vivo* studies confirmed the absence of organ abnormalities, hemolysis, or adverse immune responses, ensuring safety for clinical application. In a rat oral ulcer model, MNs outperformed traditional treatments, achieving a 90% wound healing rate in just 5 days, faster than triamcinolone acetonide ointment and other conventional therapies (Figure 17i–k). Taken together, the findings showed that encapsulating CUR and Mg-MOF to MNs synergistically improved oral ulcer treatment by combining antioxidant, anti-inflammatory, antibacterial, and prohealing properties more than conventional treatments, making them a promising oral ulcer treatment.

Another innovative approach integrated hypoxia-treated exosomes (Exos) and silver nanoparticles (AgNPs) in a light-cured composite MN patch based on bovine serum albumin methacryloyl (BSAMA) and methacrylic gelatin (GelMA) (the combination is denoted BG) as a synergistic nanotherapeutic approach for oral ulcer healing (Figure 18a–c).^183^ BG MN samples were submerged in 10 mL of artificial saliva (pH = 7.4) at 37 °C. *In vitro* degradability of BG and BG@Exos&AgNPs revealed complete degradation at 96 h. After BG loading, the Exos and AgNPs were gradually and consistently released from MNs (Figure 18d,e). Furthermore, the increased yield of exosomal proteins detected by the kit indicated the sustained release of Exos and AgNPs from the MN patch. By integrating biocompatible materials with bioactive NPs and Exos, these BG@Exos&AgNPs MN patches significantly improved cell migration and proliferation in HUVEC and human oral fibroblasts (HOrFs) in a concentration-dependent manner with Exos, peaking at 180 ng/mL, facilitating rapid tissue regeneration. The enhanced neovascularization accelerated tissue repair, with no abnormal cell proliferation, ensuring safe application. Exos regulated immune activity by reducing the levels of inflammatory cytokines IL-2 and TNF-α in LPS-stimulated macrophages. In the SD rat oral ulcer model, MN patches accelerated wound closure, outperforming other treatment groups (Figure 18f–i). Taken together, the synergistic combination of Exos and AgNPs in dissolving MNs enhanced wound healing through a multitargeted approach, including anti-inflammatory, antimicrobial, proangiogenic, and regenerative effects.

**Figure 18:**
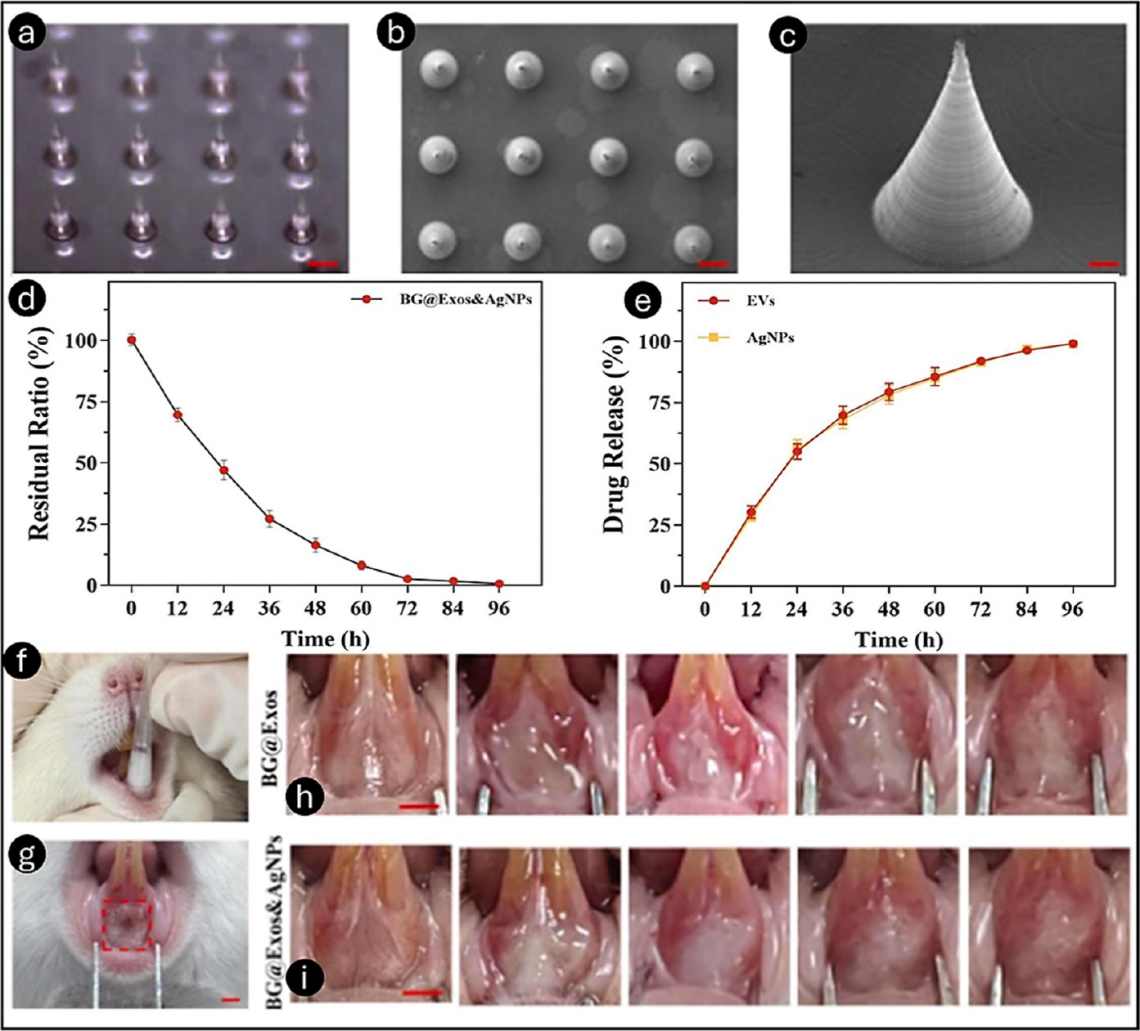
Physical representation (a) and SEM images of BG MN patches (b,c). The scale bars are 500 μm (a) and 200 μm (b,c). Degradation of BG MN patches quantified by mass loss rate (d) and release concentrations of Exos and AgNPs (e). Development of the oral ulcer model with the glacial acetic acid cauterization technique (f) and administration of BG MN patches to the oral ulcers in rats (g). Photographs depicting the progression of mouth ulcers in rats from day 0 to day 4 of treatment with BG@Exos (h) and BG@Exos&AgNPs (i). Reprinted with permission from ref 183 Copyright 2024, Elsevier.

The dissolving MN patch developed by Qu et al. represented a synergistic nanotherapeutic strategy by integrating mesoporous polydopamine nanoparticles (MPDA; 5, 10, and 20 mg) loaded with triamcinolone acetonide (TA, 2 mg/mL), HA, and *Bletilla striata* polysaccharide (BSP).^45^ With an optimal HA/BSP ratio of 2:1, the patches demonstrated efficient and rapid dissolution within 3 min after insertion into the sublingual mucosa of rats. The controlled and sustained release of TA from MPDA further optimized the therapeutic effects while reducing the required drug dosage. This combination demonstrated a synergistic reduction in the level of inflammatory cytokines, particularly TNF-α, in HOKs and hGFs cell lines. Hemolysis assays confirmed a hemolysis rate below 2%, which met safety standards. Minimal cytotoxicity was observed, with cell viability remaining above 50% even at higher concentrations. The MN insertion process caused minimal skin damage with insertion pores disappearing within 30 min, and no significant inflammatory response was observed. In a rat oral ulcer model, the MNs achieved an 88.57% wound closure rate by day 7, significantly outperforming conventional ointments despite a lower TA dosage. Histological analysis further validated reduced inflammation, enhanced collagen regeneration, and increased neovascularization, confirming their efficacy in promoting rapid oral ulcer healing. In a nutshell, the combination of MPDA-based controlled drug release, HA and BSP-mediated wound healing, and TA’s anti-inflammatory effects creates a highly effective, biocompatible, and minimally invasive therapeutic approach for oral ulcer treatment.

Collectively, these studies highlight the potential of the synergistic approach of nanoformulations and MNs, which offers a transformative paradigm for treating oral ulcers. Moreover, a coherent overview of the therapeutic potential of DMNs in oral ulcer management is depicted in Table 3. These summarized outcomes from the above-discussed case studies highlight the polymeric composites reported for the fabrication of DMNs, their efficacy in delivering different categories of therapeutic biomolecules, and geometrical configurations of DMNs, thereby underscoring their role in accelerating mucosal healing in *in vitro* and *in vivo* models. Combining the advantages of targeted drug delivery, enhanced bioavailability, and sustained release, this innovative strategy can be significantly used in the management of this common and often debilitating condition. Continued research and development in this area, including preclinical and clinical studies, are warranted to translate these promising findings into effective clinical applications.

**Table 3:** Summarized Outcomes of the DMNs from Studies on Therapeutic Delivery for Oral Ulcers

polymeric composites	encapsulated therapeutic(s)	DMNs dimensions	penetration force/mechanical strength	*in vitro* cell line	*in vivo* animal models/oral ulcer-inducing agent	time taken to dissolve MNs	refs
HA, GelMA	lidocaine, dexamethasone		mechanical strength increased proportionally with the concentration of the pregel solution	RAW 264.7 macrophages, human umbilical vein endothelial cells (HUVECs), and 3T3 cells	SD rats; acetic acid		^142^
10% (w/v) PVP	Astragalus polysaccharide	pyramid-shaped, with a height of 700 μm, and interneedle spacing of 350 μm. 400 needles (20 × 20 array) arranged uniformly on the backing layer	penetration force of 35.34 N and a compressive strength of 0.1623 MPa	human oral keratinocytes (HOKs), and RAW 264.7 macrophages, HUVECs	SD rats; 50% acetic acid	2 min	^146^
HA	dexamethasone acetate, vitamin C, and tetracaine hydrochloride	0.5 cm × 0.5 cm patch, base with an array of 10 × 10 cone-shaped, and each MN has a height of 350 μm, and a base diameter of 200 μm	16–48 μm tip displacement, which is <14% of full length, indicating sufficient mechanical integrity	rat aortic endothelial cells (RAECs)	SD rats; cotton swab immersed in 99.9% acetic acid	10 s	^56^
HA, 10% PVA	minocycline hydrochloride	square array with dimensions of 1 × 1 cm^2^, consisting of 15 × 15 needles. Conical in shape, with a length of 550 μm, a diameter of 210 μm, and a spacing of 600 μm between the needles	withstood up to 0.32 N. Conical geometry enabled efficient mucosal penetration	HUVECs, and RAW 264.7 cells	male SD rats; inoculated with 100 μL. *S. aureus* suspension at a concentration of 1 × 10^6^ cfu/mL		^180^
HA and hydroxypropyl trimethyl ammonium chloride chitosan (HACC)	dexamethasone, (DXMS) and basic fibroblast growth factor (bFGF)	square array of 400 (20 × 20) needles, each with a triangular pyramidal shape with a height of 430 μm and spacing of 700 μm	MN patch achieved an epidermal penetration depth of 400 ± 8 μm	human oral fibroblasts (HOrF), and HUVECs	SD rats; 50% glacial acetic acid	2 min	^59^
HA	betamethasone 21-phosphate sodium (BSP), and betamethasone 17,21-dipropionate (BDP)	15 × 15 arrayed MNs with a tip-to-tip space of 600 μm. Conical in shape, 300 μm in width and 700 μm in height	sufficient mechanical strength with an insertion depth of 207 ± 3 μm into the abdominal mucosa	primary human gingival fibroblasts (hGFs)	adult SD rats; 90% phenol solution	3 min	^55^
HA–PVP, PVA–ethyl cellulose-backing layer	betamethasone sodium phosphate (BSP)	pyramidal-shaped needles, arranged in a 10 × 10 array with a height of 450 μm, a bottom diameter of 190 μm, and a tip-to-tip space is about 475 μm	compression force: 0.268 N per needle at 0.3 mm displacement penetration efficiency: >97% insertion into oral mucosa confirmed by trypan blue staining	HOKs	adult SD rats	20 s	^141^
methacrylate gelatin shell layer of basic fibroblast growth factor (bFGF), HA core	dexamethasone (DXMS) and zeolite imidazoline framework-8 (ZIF-8)	conical-shaped needles, arranged in a 20 × 20 array. Each needle is 600 μm high, with a 400 μm base diameter, and 600 μm center-to-center spacing		RAW 264.7 macrophages		2 min	^50^
10% (w/v) HA	triamcinolone acetonide (TA), epidermal growth factor (EGF), and zeolitic imidazolate framework-8 (ZIF-8)	20 × 20 MN array with conical-shaped tips. Needle length was 470 ± 5 μm, and the distance between the needles was 700 ± 5 μm	sufficient mechanical strength with an insertion depth of 350 μm	HUVECs	male SD rats; 50% glacial acetic acid with a diameter of 5 mm	5 min	^54^
HA	recombinant bovine basic fibroblast growth factor (rbFGF) and cetyl pyridinium chloride (CPC)	11 × 11 array, with conical-shaped needles with a height of 564.23 ± 6.97 μm, base diameter of 281.55 ± 5.16 μm, and interneedle spacing of 576.31 ± 10.06 μm	sufficient mechanical strength with compressive stress values of 91.37 ± 4.02 kPa for rbFGF/CPC-loaded variants at 60% strain, and an insertion depth of 151.42 ± 41.53 μm	L929 mouse fibroblast cells	male SD rats; 50% acetic acid	5 min	^151^
methacrylated carboxymethyl chitosan (CMCSMA)	bone marrow mesenchymal stem cell-derived exosomes (Exos), and folic acid-magnetic nanoparticles (Fmns)	11 × 11 arrays on a 10 × 10 mm patch. Each with a length of 400 μm and a height of 800 μm	sufficient mechanical strength, MNs could withstand a force of 17 N	HOKs	male SD rats; 70% acetic acid		^182^
silk fibroin (SF)	lipopolysaccharide (LPS)-preconditioned bone marrow mesenchymal stem cells, their secreted exosomes (LPS-pre-Exos), and zeolitic imidazolate framework-8 (ZIF-8)	20 × 20 array, conical-shaped needles with a length of 700 ± 5 μm	sufficient mechanical strength, the adhesive strength of MN was 7 kPa	HUVECs, and human buccal mucosa fibroblasts (HBMFs)	male SD rats; 50% solution of glacial acetic acid		^58^
HA	curcumin loaded with porous magnesium metal–organic framework (Mg-MOF)	20 × 20 array, quadrangular pyramidal shape. 300 μm diameter at the base and 600 μm height	sufficient mechanical strength, the adhesive strength of MNs was 13 kPa	HUVECs	male SD rats; 50% glacial acetic acid	25 min	^57^
bovine serum albumin methacryloyl (BSAMA) and methacrylic gelatin (GelMA)	rat bone mesenchymal stem cells-derived exosomes and Ag nanoparticles	20 × 20 arrays, triangular pyramidal in shape, with a height of 430 μm and a spacing of 700 μm	0.58 N	HUVECs, and HOrF	SD rats; 50% glacial acetic acid		^183^
24% *Bletilla striata* polysaccharide (BSP), HA	mesoporous polydopamine nanoparticles (MPDA) loaded with triamcinolone acetonide (TA)	conical in shape, a 15 × 15 array, each with a height of 700 μm and a base diameter of 300 μm	sufficient mechanical strength, insertion depth of 418 ± 2 μm into the oral mucosa	HOKs, and human gingival fibroblasts (hGFs)	SD rats; 90% phenol solution	3 min	^45^

## 7 Challenges, Limitations, and Future Perspectives

Nanotherapeutic MNs represent a groundbreaking advancement in oral ulcer treatment, offering targeted, minimally invasive drug delivery. However, several challenges and limitations hinder their widespread acceptance. One of the primary challenges is the complex environment of the oral cavity, which presents barriers to drug retention and absorption. Continuous mucosal turnover, the presence of saliva, and mechanical forces from speaking, chewing, and swallowing can lead to premature dissolution or displacement of MNs before achieving optimal therapeutic effects. Furthermore, a major problem with many therapeutic agents is their bioavailability, especially with hydrophobic drugs like corticosteroids. Despite incorporation of nanotechnology to enhance solubility and stability, ensuring consistent and prolonged drug release in the oral cavity is still a concern. The mechanical characteristics of dissolving MNs represent yet another significant constraint. Although structural integrity and dissolution rates have improved due to advancements in polymer-based MNs, maintaining the ideal balance between adequate penetration depth and minimal discomfort is still challenging. MNs that are too rigid may cause tissue irritation, whereas flexible or rapidly dissolving MNs may not deliver drugs effectively. Furthermore, patient compliance is another hurdle, as concerns regarding safety, discomfort, and unfamiliarity with the technology may lead to a reluctance to adopt. The perception of inserting MNs into the sensitive environment of the oral cavity, even if painless, could daunt some patients, requiring further efforts in awareness.

Clinical translation is also significantly hampered by manufacturing and regulatory issues. Currently, there are no standardized guidelines for quality control, safety, and efficacy assessment of MN-based therapies in oral applications. The lack of regulatory clarity complicates the approval process, delaying the introduction of these novel treatments into mainstream healthcare. Additionally, large-scale manufacturing of nanoenhanced MNs with precise drug loading and consistent quality remains costly and technically demanding. Scaling up production while maintaining affordability and accessibility is crucial to ensuring widespread adoption. Despite these challenges, the future of nanoenhanced MNs in oral ulcer management is promising. Ongoing research is focused on optimizing nanoformulations to improve drug compatibility with dissolving MNs, enhancing their mechanical strength, and fine-tuning their dissolution rates for prolonged therapeutic effects. The development of multilayered or stimuli-responsive MNs capable of controlled drug release could further enhance efficacy and patient convenience. Integrating anti-inflammatory agents, growth factors, and antimicrobial compounds into a single MN patch may provide a comprehensive solution that not only alleviates symptoms but also addresses the underlying causes.

Moreover, advancements in smart MN technology, such as biosensing MNs that monitor pH, inflammation levels, or microbial activity in real time, could revolutionize personalized treatment approaches. The integration of wireless or wearable devices to track healing progress and adjust drug delivery accordingly could significantly enhance the patient outcomes. Collaboration among researchers, clinicians, and regulatory authorities is essential in establishing clear safety and efficacy standards to facilitate clinical adoption. Educating healthcare professionals and patients about the benefits of MN technology will also be crucial in driving acceptance. In conclusion, while nanotherapeutic MNs hold immense potential in revolutionizing oral ulcer treatment, overcoming challenges related to bioavailability, mechanical properties, manufacturing, and regulation is essential. With continued innovation and interdisciplinary collaboration, these advanced drug delivery systems could become a transformative solution for oral healthcare.

## Supplementary Material



## Data Availability

Data are available throughout the manuscript and Supporting Information.

## Notes

Table S1 summarizing the current treatment modalities employed in the management of oral ulcers; comprehensive insights into the design, mechanisms of action, and physicochemical characteristics of hydrogel, solid, hollow, and coated MNs (Section S4.2.1–S4.2.4); and additional references relevant to the discussed content (PDF)
