# Supplementary material for: Harnessing Polymeric Dissolvable Microneedles: Precision Delivery of Therapeutics for Oral Ulcers
Source: ACS Omega. 2025 Nov 19;10(47):56932–63. doi: 10.1021/acsomega.5c05654 (PMC12676321; doi:10.1021/acsomega.5c05654)
Supplement: Supplementary file 1 [file ao5c05654_si_001.pdf]

Harnessing Polymeric Dissolvable Microneedles: Precision Delivery of Therapeutics for Oral Ulcers

Maria Nison[1], Megha Kotian[1], Vasudev R Pai[1*], Sony Priyanka Bandi[2], Popat Mohite[3], Deepanjan Datta[4*]

[1]Department of Pharmacognosy, Manipal College of Pharmaceutical Sciences, Manipal Academy of Higher Education, Manipal 576 104, Karnataka State, India

[2]Department of Pharmacy, Birla Institute of Technology and Science (BITS) Pilani, Hyderabad Campus, Hyderabad 500 078, Telangana State, India

[3]Department of Chemistry, AETs St. John Institute of Pharmacy and Research, Palghar, Maharashtra 401 404, India

[4]Department of Pharmaceutics, Manipal College of Pharmaceutical Sciences, Manipal Academy of Higher Education, Manipal 576 104, Karnataka State, India

***Corresponding Author**

Deepanjan Datta, PhD
Assistant Professor
Department of Pharmaceutics
Manipal College of Pharmaceutical Sciences,
Manipal Academy of Higher Education, Manipal 576 104, Karnataka State, India
Email: deepanjan.datta@manipal.edu; deepanjandtt@gmail.com

SUPPLEMENTARY INFORMATION

Table S1. List of current treatments, their dose, dosage form, and reported limitations for oral ulcers

Current treatments	Dosage form	Limitations	Ref.
Triamcinolone acetonide	0.1% ointment (3 to 4 times per day), injection	Burning sensation, redness, drying of the skin	1,2
Fluocinolone acetonide	Gel (0.025–0.05%) 3-4x/day	Irritation, folliculitis	3
Levamisole	Tablet (50mg; 3 times/day, 150 mg total for 3 days per week)	Headache, nausea, dysgeusia, hypersomnia	4,5
Amlexanox	5% Ointment (3-4 times/day)	-	6,7
Choline salicylate + Benzalkonium chloride + Lignocaine HCl	Gel (3-4x/day)	Numbness of the tongue	8
Triclosan	Gel/ rinse/paste (3 times/day)	Banned by the FDA in 2016 due to safety concerns	9
B-complex with Vitamin C	tablet (1x/day)	-	10,11
Doxycycline	Mouth rinse (100mg in 10 ml of water 4x/day, Gargle for 2–3 minutes)	-	12

S4.2.1. Hydrogel MNs

Hydrogel MNs (HMNs) are an innovative drug delivery system that combines the minimally invasive nature of MNs with the unique properties of hydrogels. These MNs are particularly promising for treating oral ulcers, as they can deliver therapeutic agents directly to the affected mucosal tissues. A hydrogel-forming matrix is used to manufacture a relatively new kind of MN, initially reported in 2012.[13] The mechanism of action follows the "poke, swell, and release" approach. Initially, the MNs painlessly penetrate the oral mucosa, creating microchannels. Upon insertion, they absorb interstitial fluid, causing the hydrogel matrix to swell. These swelling forms continuous hydrogel microchannels, facilitating the controlled and sustained diffusion of encapsulated drugs into the surrounding tissue.[14] This method ensures effective therapeutic delivery while minimizing discomfort and avoiding damage to underlying tissues. Materials used for fabricating these HMNs, such as biodegradable polymers like HA, polymethyl vinyl ether-co-maleic acid (PMVE/MA), and chitosan, are chosen for their biocompatibility and ability to dissolve safely within the oral environment.[15] Their ability to adhere to the moist and dynamic environment of the oral cavity enhances drug retention at the site of the ulcer, potentially improving therapeutic outcomes. Despite these benefits, challenges remain in optimizing hydrogel MNs for oral applications, such as ensuring sufficient mechanical strength to penetrate the mucosal tissue and achieving adequate drug loading capacity.[16] Ongoing research is focused on addressing these issues to fully realize the potential of HMNs in the management of oral ulcers.

S4.2.2. Solid MNs

Solid MNs (SMNs) are a foundational type of MN system utilized to enhance drug delivery across biological barriers such as the skin and mucosal tissues.[17] In the context of oral ulcer treatment, solid MNs serve as a mechanical pre-treatment method to facilitate the absorption of therapeutic agents. The "poke and patch" technique involves two steps: first, these MNs are applied to the oral mucosa to create transient microchannels by puncturing the outer tissue layer by passive diffusion; second, a drug-containing formulation (e.g., gel, cream, or patch) is applied over the treated area. These microchannels enhance the permeability of the tissue, allowing the drug to diffuse more effectively into the underlying layers.[18] Solid MNs are typically

fabricated from materials such as stainless steel, titanium, silicon, or durable polymers. These materials are chosen for their mechanical strength and biocompatibility, ensuring effective tissue penetration without causing significant discomfort.[19] In the treatment of oral ulcers, the application of these MNs can enhance the delivery of therapeutic agents directly to the lesion site. By creating microchannels in the oral mucosa, they facilitate increased drug permeability, potentially leading to improved healing outcomes. However, the "poke and patch" approach has certain limitations; the microchannels created tend to close within a short period, potentially limiting the window for effective drug absorption. Additionally, the two-step application process may introduce variability in dosing and require careful coordination to ensure optimal therapeutic efficacy.[20] Despite these challenges, SMNs offer a promising method for enhancing drug delivery in various medical applications, including the treatment of oral ulcers. Ongoing research aims to optimize their design and application techniques to maximize therapeutic efficacy.

S4.2.3. Hollow MNs

Hollow microneedles (HMNs) are a specialized type of MN designed for the direct delivery of liquid drugs into the skin or mucosal tissues.[21] Unlike SMNs, they feature an internal bore or lumen (5–70 μm wide) that enables the passive diffusion of drugs or active delivery via external pressure from syringes, pumps, or gas.[22] The "poke and flow" mechanism involves inserting HMNs into the tissue to create microchannels, through which the drug solution flows from an attached reservoir into the targeted epidermal or dermal layers.[23] HMNs excel in delivering larger drug volumes (typically 10–100 μL/min) and high molecular weight substances, making them ideal for liquid vaccines, insulin, and other biologics.[24] Their design allows for precise control over drug release kinetics, which can be further modulated by integrating microfluidic chips or micropumps within the MN array. Compared to SMNs, HMNs release significantly higher amounts of bioactive molecules and offer advantages such as cost-effectiveness, ease of manufacturing, and tunable release profiles, ranging from burst release to sustained delivery over days to weeks.[25] HMNs are typically fabricated from silicon, metals, or polymers, among others.[26] Their release kinetics can be adjusted by modifying material formulations and production conditions. Like hypodermic needles, HMNs permit flow rate and pressure modulation, enabling tailored drug delivery. Over the years, they have been successfully used for vaccinations and inoculations,

demonstrating their clinical utility.[27] Despite their benefits, they are mechanically weaker than SMNs and require careful design optimization to prevent structural failure during insertion. Technical challenges include lumen clogging and drug leakage during injection, which remain active areas of research. These limitations have historically reduced their commercial appeal compared to SMNs, though ongoing advancements aim to address these issues.

S4.2.4. Coated MNs

The coated microneedles (CMNs) are a type of SMN coated with a drug solution. Depending on the thickness of the coating layer, it often delivers a small amount of the drug solution. The success of delivering a drug using these MNs depends on the ability to coat a drug layer onto MNs effectively.[28] A coated MN can be used to deliver proteins and DNA in a minimally invasive manner.[29] An advantage of a CMN is the rapid delivery of the drug to the target site; however, the residual drug at the tip of the needle can infect other patients. CMNs rapidly deliver macromolecules such as proteins, peptides,[30] DNA,[31] and vaccines.[32] The primary disadvantage of this form of MN is the limited amount of drug solution that can be coated on the MNs' surface, even though it allows for a simple one-step application procedure. The use of CMNs is, therefore, restricted to potent molecules or drugs.

References

(1) Rashid, S. Al; Rahman, S. Z.; Khan, S.; Rashid, S. Al; Rahman, S. Z.; Khan, S. Comparative Study for Safety and Efficacy of a Novel Folic Acid Formulation Gel with 0.1% Triamcinolone Acetonide Paste in Experimentally Induced Oral Ulcer. *Indian J Physiol Pharmacol* **2021**, *65* (1), 39–44. https://doi.org/10.25259/IJPP_11_2021.

(2) Richards, R. N. Side Effects of Short-Term Oral Corticosteroids. *J Cutan Med Surg* **2008**, *12* (2), 77–81. https://doi.org/10.2310/7750.2008.07029.

(3) Liu, C.; Zhou, Z.; Liu, G.; Wang, Q.; Chen, J.; Wang, L.; Zhou, Y.; Dong, G.; Xu, X.; Wang, Y.; Guo, Y.; Lin, M.; Wu, L.; Du, G.; Wei, C.; Zeng, X.; Wang, X.; Wu, J.; Li, B.; Zhou, G.; Zhou, H. Efficacy and Safety of Dexamethasone Ointment on Recurrent Aphthous Ulceration. *Am J Med* **2012**, *125* (3), 292–301. https://doi.org/10.1016/J.AMJMED.2011.09.011.

(4) Scheinfeld, N.; Rosenberg, J. D.; Weinberg, J. M. Levamisole in Dermatology: A Review. *Am J Clin Dermatol* **2004**, *5* (2), 97–104. https://doi.org/10.2165/00128071-200405020-00004/FIGURES/TAB1.

(5) Sun, A.; Chiang, C. -P; Chiou, P. -S; Wang, J. -T; Liu, B. -Y; Wu, Y. -C. Immunomodulation by Levamisole in Patients with Recurrent Aphthous Ulcers or Oral Lichen Planus. *Journal of Oral Pathology & Medicine* **1994**, *23* (4), 172–177. https://doi.org/10.1111/J.1600-0714.1994.TB01108.X.

(6) Bell, J. Amlexanox for the Treatment of Recurrent Aphthous Ulcers. *Clin Drug Investig* **2005**, *25* (9), 555–566. https://doi.org/10.2165/00044011-200525090-00001/FIGURES/TAB4.

(7) Darshan, D. D.; Kumar, C. N. V.; Kumar, A. D. M.; Manikantan, N. S.; Balakrishnan, D.; Uthkal, M. P. Clinical Study to Know the Efficacy of Amlexanox 5% with Other Topical Antiseptic, Analgesic and Anesthetic Agents in Treating Minor RAS. *J Int Oral Health* **2014**, *6* (1), 5.

(8) Editor, G.; Endres, F.; Ueno, K.; Tokuda, H.; Watanabe, M.; Yoshida, Y.; Saito, G.; Wood, N.; Stephens, G.; MacFarlane, D. R.; Pringle, J. M.; Howlett, P. C.; Forsyth, M.; Hayes, R.; Warr, G. G.; Atkin, R.; Chem, P.; Liu, H.; Liu, Y.; Li, J.; Chem Chem, P.; Höfft, O.; Borisenko, N.; Henrique Gasparotto, L.; Prowald, A.; Al-Salman, R.; Carstens, T.; Bund, A.; Zein El Abedin, S.; Brettholle, M.; Höfft, O.; Klarhöfer, L.; Mathes, S.; Maus-Friedrichs, W.; Zein El Abedin, S.; Krischok, S.; Janek, J.; Kameyama, T.; Ohno, Y.; Kurimoto, T.; Okazaki, K.; Uematsu, T.; Kuwabata, S.; Krekeler, C.; Dommert, F.; Schmidt, J.; Zhao, Y. Y.; Holm, C.; Berger, R.; Delle, L.; Min Lee, J.; Palgunadi, J.; Hyung Kim, J.; Jung, S.; Choi, Y.; Cheong, M.; Sik Kim, H.; Abbott, A. P.; Qiu, F.; A Abood, H. M.; Rostom Ali, M.; Ryder, K. S.; M Martindale, B. C.; Ward Jones, S. E.; Glyn Jones, S.; Man Yau, H.; Davies, E.; Hook, J. M.; A Youngs, T. G.; Harper, J. B.; Croft, A. K.; Cui, Y.; Biondi, I.; Chaubey, M.; Yang, X.; Fei, Z.; Scopelliti, R.; Hartinger, C. G.; Li, Y.; Chiappe, C.; Dyson, P. J.; Lunstroot, K.; Driesen, K.; Nockemann, P.; Viau, L.; Hubert Mutin, P.; Vioux, A.; Binnemans, K.; Hayaki,

S.; Kido, K.; Sato, H.; Sakaki, S.; Strehmel, V.; Rexhausen, H.; Strauch, P.; Sato, B. M.; de Oliveira, C. G.; Martins, C. T.; El, O. A. In Search of Pure Liquid Salt Forms of Aspirin : Ionic Liquid Approaches with Acetylsalicylic Acid and Salicylic Acid. *Physical Chemistry Chemical Physics* **2010**, *12* (8), 2011–2017. https://doi.org/10.1039/B923855G.

(9) Satheeshkumar, P. S.; Chamba, M. S.; Balan, A.; Sreelatha, K. T.; Bhatathiri, V. N.; Bose, T. Effectiveness of Triclosan in the Management of Radiation-Induced Oral Mucositis: A Randomized Clinical Trial. *J Cancer Res Ther* **2010**, *6* (4), 466–472. https://doi.org/10.4103/0973-1482.77109.

(10) Liu, H. L.; Chiu, S. C. The Effectiveness of Vitamin B12 for Relieving Pain in Aphthous Ulcers: A Randomized, Double-Blind, Placebo-Controlled Trial. *Pain Management Nursing* **2015**, *16* (3), 182–187. https://doi.org/10.1016/J.PMN.2014.06.008.

(11) Sun, H.; Gao, J.; Li, D.; Li, B. Observations on the Clinical Efficacy of RhaFGF Combined with Vitamin B Complex for Patients with Severe Recurrent Aphthous Ulcer. *Pak J Med Sci* **2021**, *37* (7), 2004. https://doi.org/10.12669/PJMS.37.7.4364.

(12) Preshaw, P. M.; Grainger, P.; Bradshaw, M. H.; Mohammad, A. R.; Powala, C. V.; Nolan, A. Subantimicrobial Dose Doxycycline in the Treatment of Recurrent Oral Aphthous Ulceration: A Pilot Study. *Journal of Oral Pathology & Medicine* **2007**, *36* (4), 236–240. https://doi.org/10.1111/J.1600-0714.2007.00507.X.

(13) Turner, J. G.; White, L. R.; Estrela, P.; Leese, H. S. Hydrogel-Forming Microneedles: Current Advancements and Future Trends. *Macromol Biosci* **2021**, *21* (2), 2000307. https://doi.org/10.1002/MABI.202000307;WGROUP:STRING:PUBLICATIO N.

(14) Li, Y.; Bi, D.; Hu, Z.; Yang, Y.; Liu, Y.; Leung, W. K. Hydrogel-Forming Microneedles with Applications in Oral Diseases Management. *Materials* **2023**, *16* (13), 4805. https://doi.org/10.3390/MA16134805.

(15) Mohite, P.; Puri, A.; Munde, S.; Ade, N.; Kumar, A.; Jantrawut, P.; Singh, S.; Chittasupho, C. Hydrogel-Forming Microneedles in the Management of Dermal Disorders Through a Non-Invasive Process: A Review. *Gels 2024, Vol. 10, Page 719* **2024**, *10* (11), 719. https://doi.org/10.3390/GELS10110719.

(16) Aroche, A. F.; Nissan, H. E.; Daniele, M. A. Hydrogel-Forming Microneedles and Applications in Interstitial Fluid Diagnostic Devices. *Adv Healthc Mater* **2024**, *14* (1), 2401782. https://doi.org/10.1002/ADHM.202401782;WGROUP:STRING:PUBLICATI ON.

(17) Ferreira, L. E. N.; Franz-Montan, M.; Benso, B.; Gill, H. S. Microneedles for Oral Mucosal Delivery – Current Trends and Perspective on Future Directions. *Expert Opin Drug Deliv* **2023**, *20* (9), 1251–1265. https://doi.org/10.1080/17425247.2023.2264189.

(18) Oliveira, C.; Teixeira, J. A.; Oliveira, N.; Ferreira, S.; Botelho, C. M. Microneedles' Device: Design, Fabrication, and Applications. *Macromol 2024, Vol. 4, Pages 320-355* **2024**, *4* (2), 320–355. https://doi.org/10.3390/MACROMOL4020019.

(19) Tariq, N.; Ashraf, M. W.; Tayyaba, S. A Review on Solid Microneedles for Biomedical Applications. *Journal of Pharmaceutical Innovation 2021 17:4* **2021**, *17* (4), 1464–1483. https://doi.org/10.1007/S12247-021-09586-X.

(20) Rzhevskiy, A. S.; Singh, T. R. R.; Donnelly, R. F.; Anissimov, Y. G. Microneedles as the Technique of Drug Delivery Enhancement in Diverse Organs and Tissues. *Journal of Controlled Release* **2018**, *270*, 184–202. https://doi.org/10.1016/J.JCONREL.2017.11.048.

(21) Qiu, H.; Gong, L.; Slezak, P.; He, S.; Lu, F.; Yu, K.; Xie, J.; Geng, Z.; Hu, E.; Zhou, Z.; Lan, G.; Xie, R. Magnetic-Navigable Silk Fibroin Microneedles for Oral Drug Delivery: Ensuring Long-Lasting Helicobacter Pylori Eradication and Rapid Hemostasis in the Stomach. *Int J Biol Macromol* **2024**, *275*, 133584. https://doi.org/10.1016/J.IJBIOMAC.2024.133584.

(22) Liu, Y.; Mao, R.; Han, S.; Yu, Z.; Xu, B.; Xu, T. Polymeric Microneedle Drug Delivery Systems: Mechanisms of Treatment, Material Properties, and Clinical Applications—A Comprehensive Review. *Polymers 2024, Vol. 16, Page 2568* **2024**, *16* (18), 2568. https://doi.org/10.3390/POLYM16182568.

(23) Ye, R.; Yang, J.; Li, Y.; Zheng, Y.; Yang, J.; Li, Y.; Liu, B.; Jiang, L. Fabrication of Tip-Hollow and Tip-Dissolvable Microneedle Arrays for Transdermal Drug Delivery. *ACS Biomater Sci Eng* **2020**, *6* (4), 2487–2494. https://doi.org/10.1021/ACSBIOMATERIALS.0C00120/ASSET/IMAGES/LARGE/AB0C00120_0002.JPEG.

(24) Zhao, J.; Xu, G.; Yao, X.; Zhou, H.; Lyu, B.; Pei, S.; Wen, P. Microneedle-Based Insulin Transdermal Delivery System: Current Status and Translation Challenges. *Drug Delivery and Translational Research 2021 12:10* **2021**, *12* (10), 2403–2427. https://doi.org/10.1007/S13346-021-01077-3.

(25) Omidian, H.; Dey, S.; Barry, C.; Silverman, J. Multifunctional Hydrogel Microneedles (HMNs) in Drug Delivery and Diagnostics. *Gels 2025, Vol. 11, Page 206* **2025**, *11* (3), 206. https://doi.org/10.3390/GELS11030206.

(26) Anbazhagan, G.; Suseela, S. B.; Sankararajan, R. Hollow Microneedles: From Materials to Market—a Comprehensive Review. *Microsystem Technologies* **2025**, 1–20. https://doi.org/10.1007/S00542-025-05854-9/FIGURES/3.

(27) Khaled Aldawood, F.; Andar, A.; Desai, S.; Giammona, G.; Fabiola Craparo, E. A Comprehensive Review of Microneedles: Types, Materials, Processes, Characterizations and Applications. *Polymers 2021, Vol. 13, Page 2815* **2021**, *13* (16), 2815. https://doi.org/10.3390/POLYM13162815.

(28) Ingrole, R. S. J.; Gill, H. S. Microneedle Coating Methods: A Review with a Perspective. *J Pharmacol Exp Ther* **2019**, *370* (3), 555–569. https://doi.org/10.1124/JPET.119.258707.

(29) Wu, Y.; Hutton, A. R. J.; Pandya, A. K.; Patravale, V. B.; Donnelly, R. F. Microneedle and Polymeric Films: Delivery of Proteins, Peptides and Nucleic Acids. *Handb Exp Pharmacol* **2024**, *284*, 93–111. https://doi.org/10.1007/164_2023_653/FIGURES/3.

(30) Kapoor, Y.; Milewski, M.; Dick, L.; Zhang, J.; Bothe, J. R.; Gehrt, M.; Manser, K.; Nissley, B.; Petrescu, I.; Johnson, P.; Burton, S.; Moseman, J.; Hua, V.; Grunewald, T.; Tomai, M.; Smith, R. Coated Microneedles for Transdermal Delivery of a Potent Pharmaceutical Peptide. *Biomed Microdevices* **2020**, *22* (1), 1–10. https://doi.org/10.1007/S10544-019-0462-1/FIGURES/5.

(31) Gill, H. S.; Prausnitz, M. R. Coated Microneedles for Transdermal Delivery. *Journal of Controlled Release* **2007**, *117* (2), 227–237. https://doi.org/10.1016/J.JCONREL.2006.10.017.

(32) Kwon, K. M.; Lim, S. M.; Choi, S.; Kim, D. H.; Jin, H. E.; Jee, G.; Hong, K. J.; Kim, J. Y. Microneedles: Quick and Easy Delivery Methods of Vaccines. *Clin Exp Vaccine Res* **2017**, *6* (2), 156–159. https://doi.org/10.7774/CEVR.2017.6.2.156.
